# Supplementary material for: Stereoisomeric engineering of aggregation-induced emission photosensitizers towards fungal killing
Source: Nat Commun. 2022 Nov 17;13:7046. doi: 10.1038/s41467-022-34358-7 (PMC9672067; doi:10.1038/s41467-022-34358-7)
Supplement: Supplementary file 1 — Supplementary Information [file 41467_2022_34358_MOESM1_ESM.docx]

Supplementary Information

Stereoisomeric Engineering of Aggregation-Induced Emission Photosensitizers towards Fungal Killing

Wenping Zhu,^1^ **^†^** Ying Li,^2^ **^†^** Shaoxun Guo,^1^ Wu-Jie Guo,^1^ Tuokai Peng,^1^ Hui Li,^1^ Bin Liu,^1^ Hui-Qing Peng,^1,4^* and Ben Zhong Tang^3^*

[1] Beijing Advanced Innovation Center for Soft Matter Science and Engineering, State Key Laboratory of Chemical Resource Engineering Beijing University of Chemical Technology, Beijing 100029, China

[2] Guangzhou Municipal and Guangdong Provincial Key Laboratory of Molecular Target & Clinical Pharmacology, School of Pharmaceutical Sciences, Guangzhou Medical University, Guangzhou 511436, China

[3] School of Science and Engineering, Shenzhen Institute of Aggregate Science and Technology, The Chinese University of Hong Kong Shenzhen, Guangdong 518172, China

[4] Guangdong Provincial Key Laboratory of Luminescence from Molecular Aggregates, South China University of Technology, Guangzhou 510640, China

*Corresponding Author: [hqpeng@mail.buct.edu.cn](mailto:hqpeng@mail.buct.edu.cn) (Hui-Qing Peng);
[tangbenz@cuhk.edu.cn](mailto:tangbenz@cuhk.edu.cn).(Ben Zhong Tang)

**Materials and Instruments.**

All reagents were purchased from commercial suppliers (Energy Chemical, Sigma-Aldrich, TCI) and used without further purification.^1^H NMR and ^13^C NMR spectra were measured on a Bruker Advance ΙΙΙ (400 MHz) instrument using Chloroform-D (CDCl_3_)and Deuterium Oxide (D_2_O)and Dimethyl Sulfoxide-D6 (DMSO-*d*_6_)as the solvent and tetramethylsilane as the internal reference. X-Ray single-crystal diffraction data were collected on a Gemini E X-ray diffraction (Agilent, Oxford) with graphite-monochromator Mo-Kα (λ = 0.71073 Å) at 110 K. UV-vis absorption spectra were measured by a SHIMADZU UV-2600i spectrophotometer. The photoluminescence spectra were measured by a SHIMADZU RF-600 spectro fluorophotometer. ROS assays were conducted by using a Xenon lamp (Microsolar300, Beijing Perfectlight). The quantum efficiencies were measured using an Edinburgh FS5 fluorescence spectrophotometer. Dynamic light scattering (DLS) investigations were carried out with a Malvern Zetasizer Nano ZS90 (Malvern Instruments, UK). ESR analysis was performed on a Bruker E 500 spectrometer. The lifetime of triplet states was determined on Edinburgh LP 980 spectrometer. High resolution Scanning electron microscope (HR-SEM) images were obtained using a Thermo APREOS instrument. The fluorescence images were taken by a confocal laser scanning microscope (CLSM, ZEISS-LSM900).

**Syntheses of compounds**

**Synthesis of TPE-2Br.** The synthetic procedures of 1,2-bis(4-bromophenyl)-1,2-diphenylethene (TPE-2Br) was reported in literature.^1^ To a mixture of 4,4-dibromobenzophenone (0.85 g, 2.5 mmol), benzophenone (0.45 g, 2.5 mmol) and Zn (1.95 g, 30.0 mmol) in 100 mL dry THF was added dropwise TiCl_4_ (1.65 mL, 15.0 mmol) under N_2_ at -78 °C. The mixture was stirred at -78 °C for 30 min and another 2 h at room temperature Then the reaction mixture was refluxed overnight. After cooling down to roomtemperature,10% K_2_CO_3_ (aq) was added and the mixture was filtered. The filtrates were extracted with dichloromethane and the extracts were washed with brine. After solvent removal under reduced pressure, the crude product was purified by column chromatography on silica gel using n-hexane/dichloromethane (5:1, v/v) as eluent to afford TPE-2Br as white solid (0.95 g, 78 % yield). ^1^H NMR (400 MHz, CDCl_3_): δ= 7.26 (d, J = 8.5 Hz, 4H), 7.16 (t, 6H), 7.05 - 7.03 (m, 4H), 6.91 (d, J = 8.5 Hz, 4H).

**Synthesis of the precursors of** **(*Z*)-TPE-EPy and (*E*)-TPE-EPy.** Tris-o-tolylphosphine (100 mg, 20%) and Palladium acetate (35 mg, 10%) and were introduced into a dry and degassed triethylamine / *N*, *N*-Dimethylformamide (15 mL, v/v= 10:5) mixture, which was stirred for 15 min. The mixture was degassed again by gentle bubbling of nitrogen, and TPE-2Br (555 mg,1.0 equiv) and 4-vinylpyridine (215 μL, 1.3 equiv) were added. The system was stirred at 90 ^o^C under nitrogen for 8 h. The resulting mixture was allowed to cool to room temperature, and triethylamine was removed in vacuo. The crude mixture was diluted with dichloromethane and washed with water and saturated sodium bicarbonate, after concentration, the residue was purified by column chromatography (ethyl acetate / petroleum ether (60-90 ℃) 1:50 to 2:1 with 1% triethylamine). (*Z*)-TPE-DPy was obtained in yield of 36%, then (*E*)-TPE-DPy was obtained in yield of 28%. (*Z*)-TPE-DPy ^1^H NMR (400 MHz, CDCl_3_): δ= 8.57 (d, J = 5.5 Hz, 4H), 7.51 (d, J = 5.9 Hz, 4H), 7.38 - 7.29 (m, 6H), 7.16 - 7.08 (m, 10H), 7.07 - 6.96 (m, 6H). (*E*)-TPE-DPy ^1^H NMR (400 MHz, CDCl_3_): δ= 8.58 (d, *J* = 6.1 Hz, 4H), 7.44 (d, *J* = 6.1 Hz, 4H), 7.35-7.28 (m, 6H), 7.21-7.15 (m, 6H), 7.13 -7.05 (m, 8H), 6.98 (d, *J* = 16.3 Hz, 2H).

**Synthesis of (*Z*)-TPE-EPy.** Into a 50 mL two-necked round-bottom flask was placed (*Z*)-TPE-DPy (108 mg, 0.25 mmol). The flask was evacuated under vacuum and flushed with dry nitrogen three times. After acetone (20 mL) was added and stirred for 10 min, a large excess of iodomethane (1mL) was injected dropwise via a hypodermic syringe. The mixture was allowed to react at 65 ^o^C for 6 h and deep red precipitate was formed. The red precipitate was collected by filtration and washed with dichloromethane for three times, then dried under vacuum at 40 ℃ to a constant weight. *(Z)*-TPE-EPy (red powder) was obtained in yield of 90%. (*Z*)-TPE-EPy ^1^H NMR (400 MHz, DMSO-*d*_6_) :δ=8.84 (d, *J* = 6.7 Hz, 4H), 8.17 (d, *J* = 6.7 Hz, 4H), 7.92 (d, *J* = 16.3 Hz, 2H), 7.56 (d, *J* = 8.3 Hz, 4H), 7.45 (d, *J* = 16.3 Hz, 2H), 7.24-7.15 (m, 6H), 7.13 (d, *J* = 8.2 Hz, 4H), 7.03-7.01 (m, 4H), 4.25 (s, 6H). ^13^C NMR (101MHz, DMSO-*d*_6_): δ=145.72, 145.54, 140.47, 131.98, 131.19, 128.47, 128.26, 127.46, 123.91, 123.78, 47.42. HRMS (MALDI-TOF): m/z ([C_42_H_36_N_2_]^2+^) = 284.1436; calcd = 284.1434.

**Synthesis of (*E*)-TPE-EPy.** Into a 50 ml two-necked round-bottom flask was placed (*E*)-TPE-DPy (108 mg, 0.25 mmol). The flask was evacuated under vacuum and flushed with dry nitrogen three times. After acetone (20 mL) was added and stirred for 10 min, a large excess of iodomethane (1mL) was injected dropwise via a hypodermic syringe. The mixture was allowed to react at 65^o^C for 6 h and yellow precipitate was formed. The red precipitate was collected by filtration and washed with dichloromethane for three times, then dried under vacuum at 40^o^C to a constant weight. *(E)*-TPE-EPy (yellow powder) was obtained in yield of 86%. (*E*)-TPE-EPy ^1^H NMR (400 MHz, DMSO-*d*_6_): δ=8.85 (d, *J* = 6.8 Hz, 4H), 8.18 (d, *J* = 6.8 Hz, 4H), 7.91 (d, *J* = 16.3 Hz, 2H), 7.54 (d, *J* = 8.3 Hz, 4H), 7.44 (d, *J* = 16.3 Hz, 2H), 7.23-7.17 (m, 6H), 7.11-7.01 (m, 8H), 4.25 (s, 6H). ^13^C NMR (101 MHz, DMSO-*d*_6_) δ =152.83, 145.66, 145.52, 143.05, 141.39, 140.48, 133.96, 131.89, 131.26, 128.60, 128.16, 127.60, 123.92, 123.73, 47.42. HRMS (MALDI-TOF): m/z ([C_42_H_36_N_2_]^2+^) = 284.1435; calcd = 284.1434.

**Supplementary Figure 1.** Synthetic route to the precursor of (*Z*)-TPE-EPy and (*E*)-TPE-EPy.

**Supplementary Figure 2.** Synthetic route to (*Z*)-TPE-EPy and (*E*)-TPE-EPy.


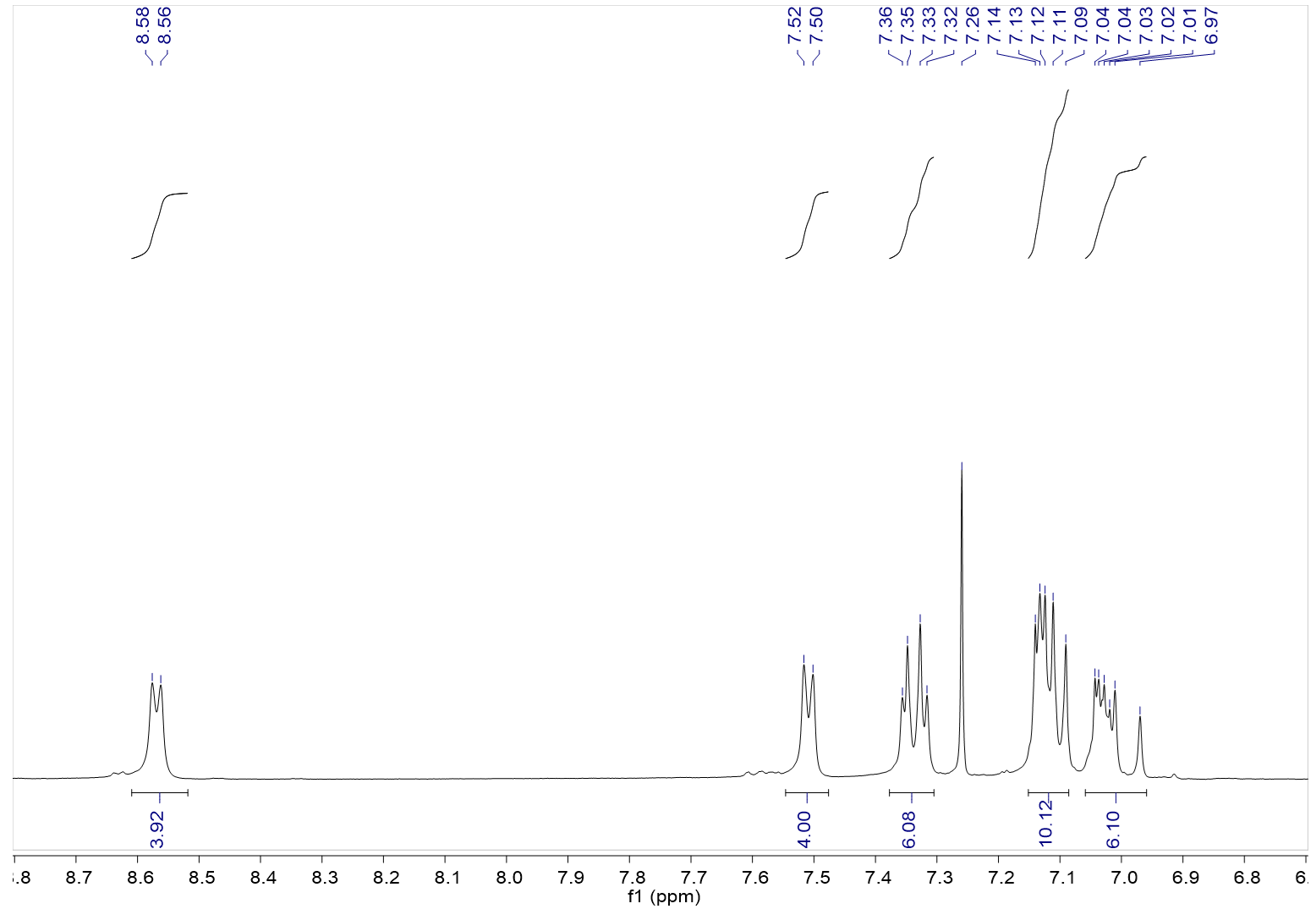


**Supplementary Figure 3.** ^1^H NMR spectrum of the precursor of (*Z*)-TPE-EPy in CDCl_3_.


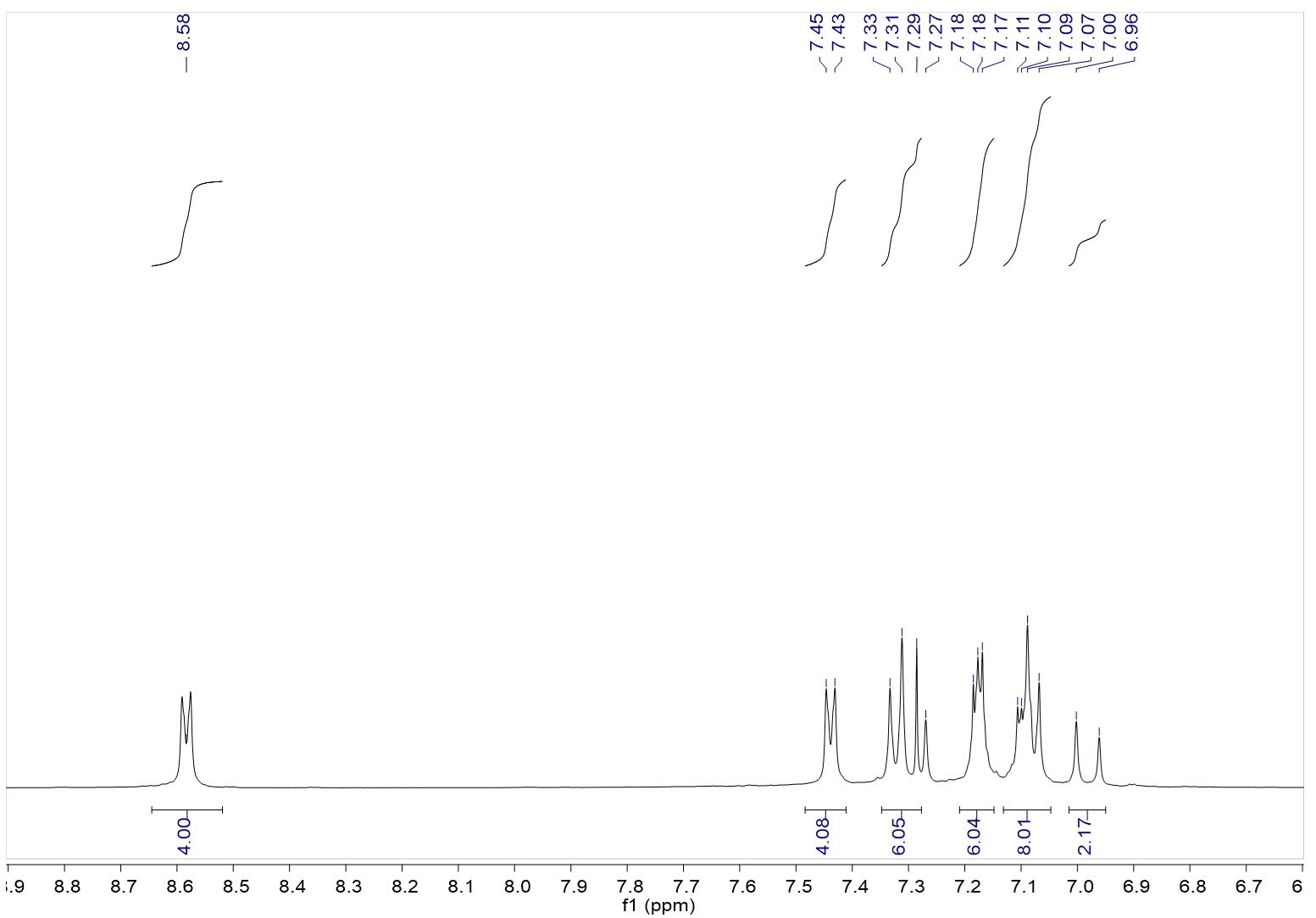


**Supplementary Figure 4.** ^1^H NMR spectrum of the precursor of (*E*)-TPE-EPy in CDCl_3_.


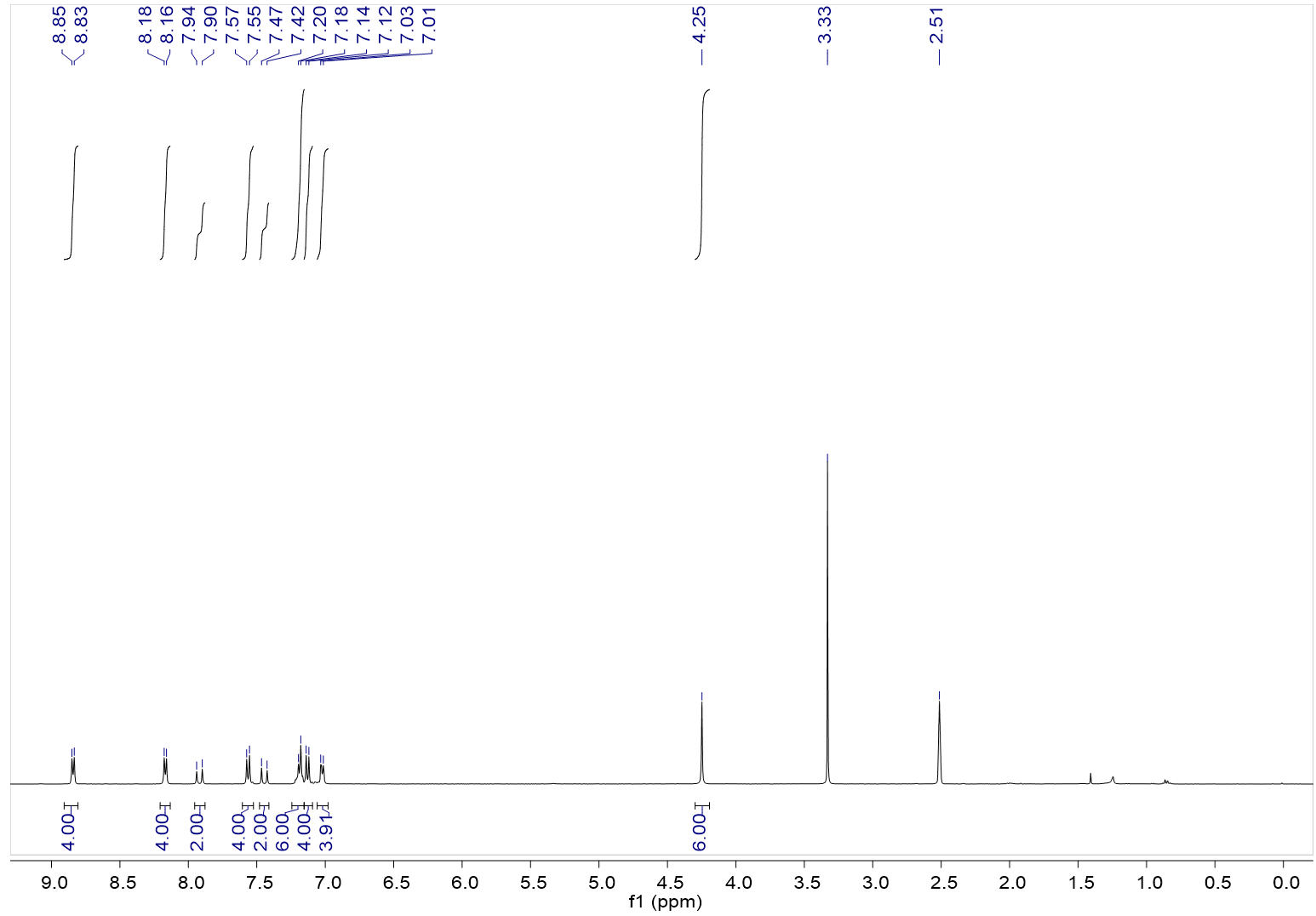


**Supplementary Figure 5.** ^1^H NMR spectrum of (*Z*)-TPE-EPy in DMSO-*d*_6_.


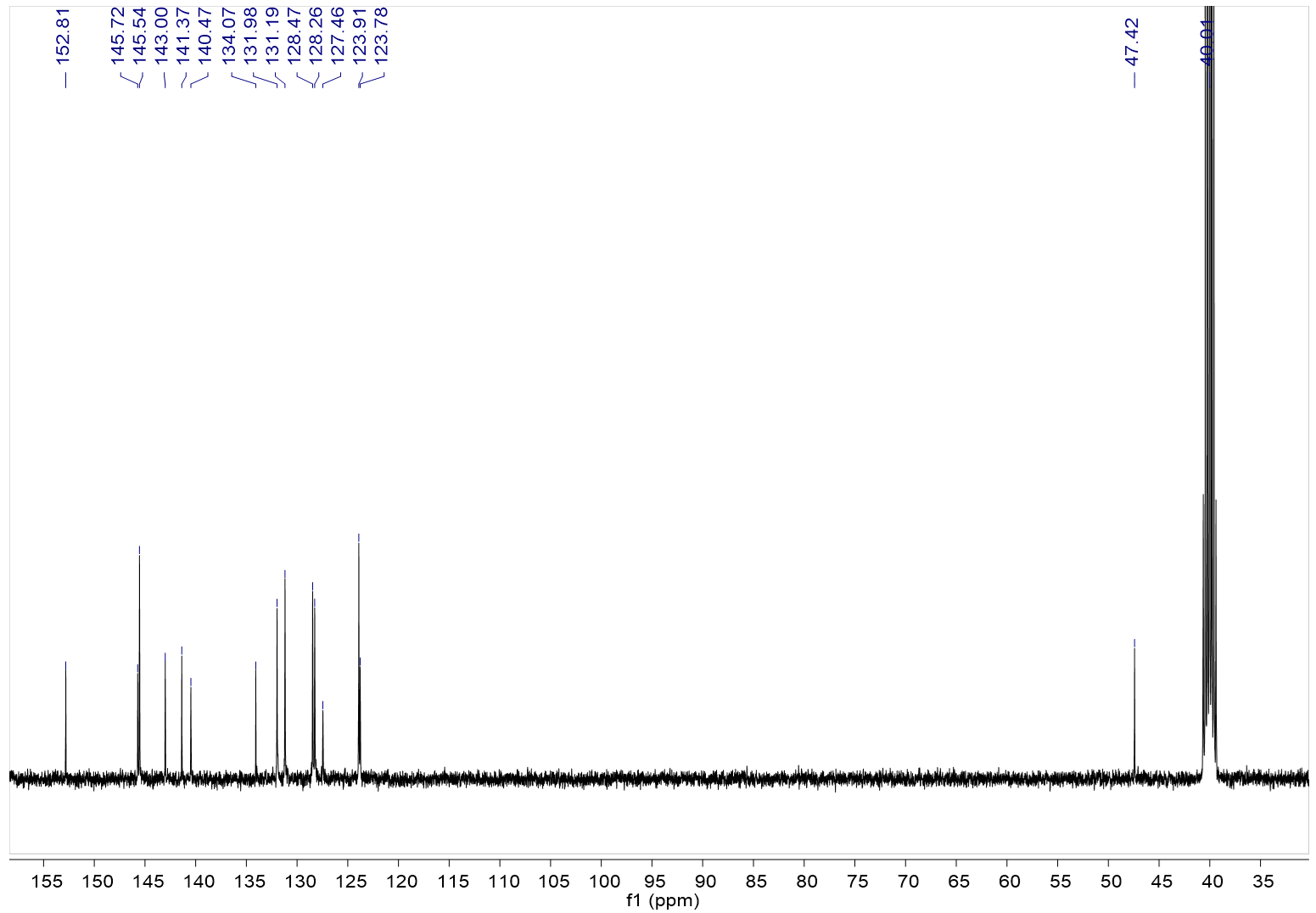


**Supplementary Figure 6.** ^13^C NMR spectrum of (*Z*)-TPE-EPy in DMSO-*d*_6_.


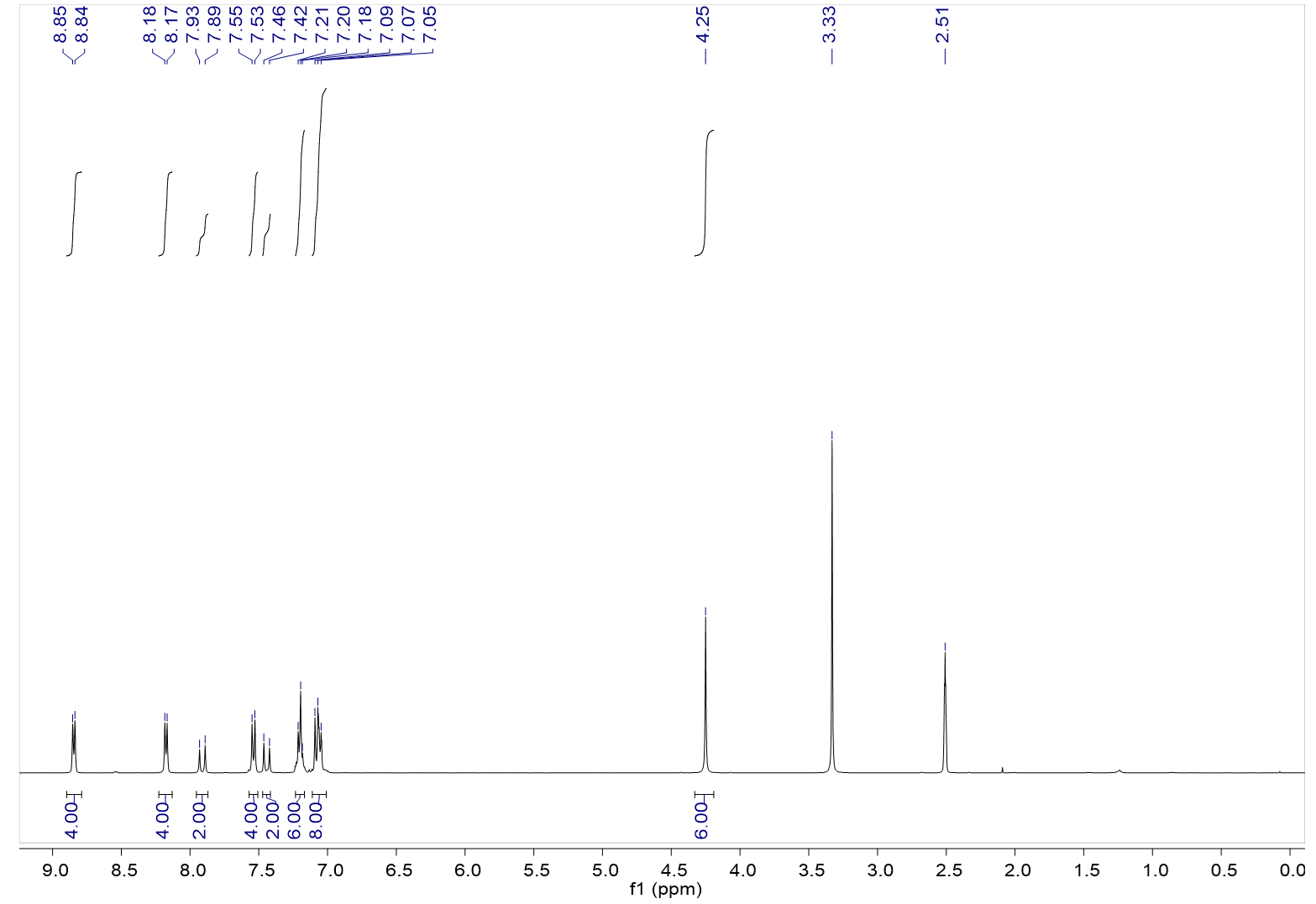


**Supplementary Figure 7.** ^1^H NMR spectrum of (*E*)-TPE-EPy in DMSO-*d*_6_.


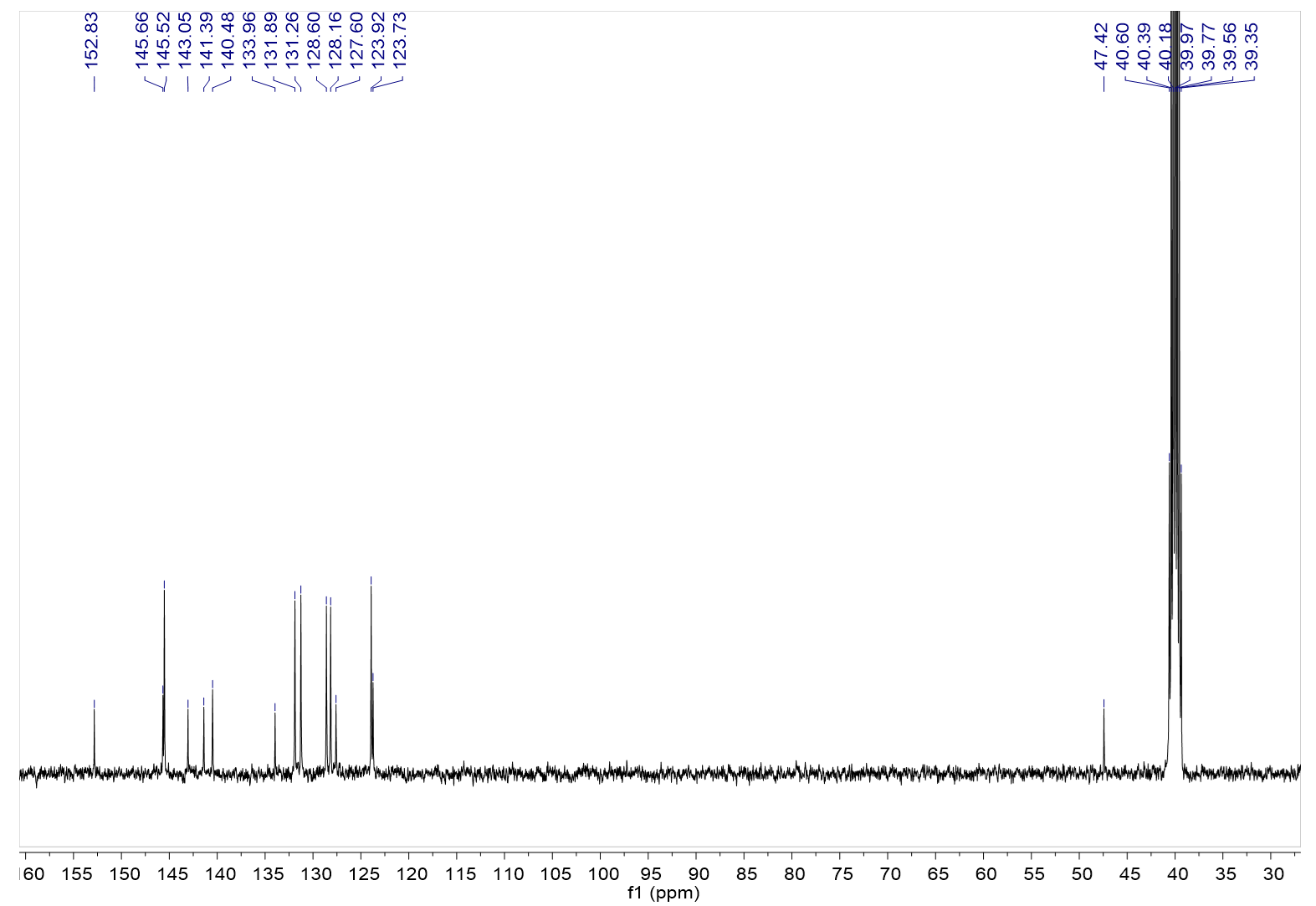


**Supplementary Figure 8.** ^13^C NMR spectrum of (*E*)-TPE-EPy in DMSO-*d*_6_.


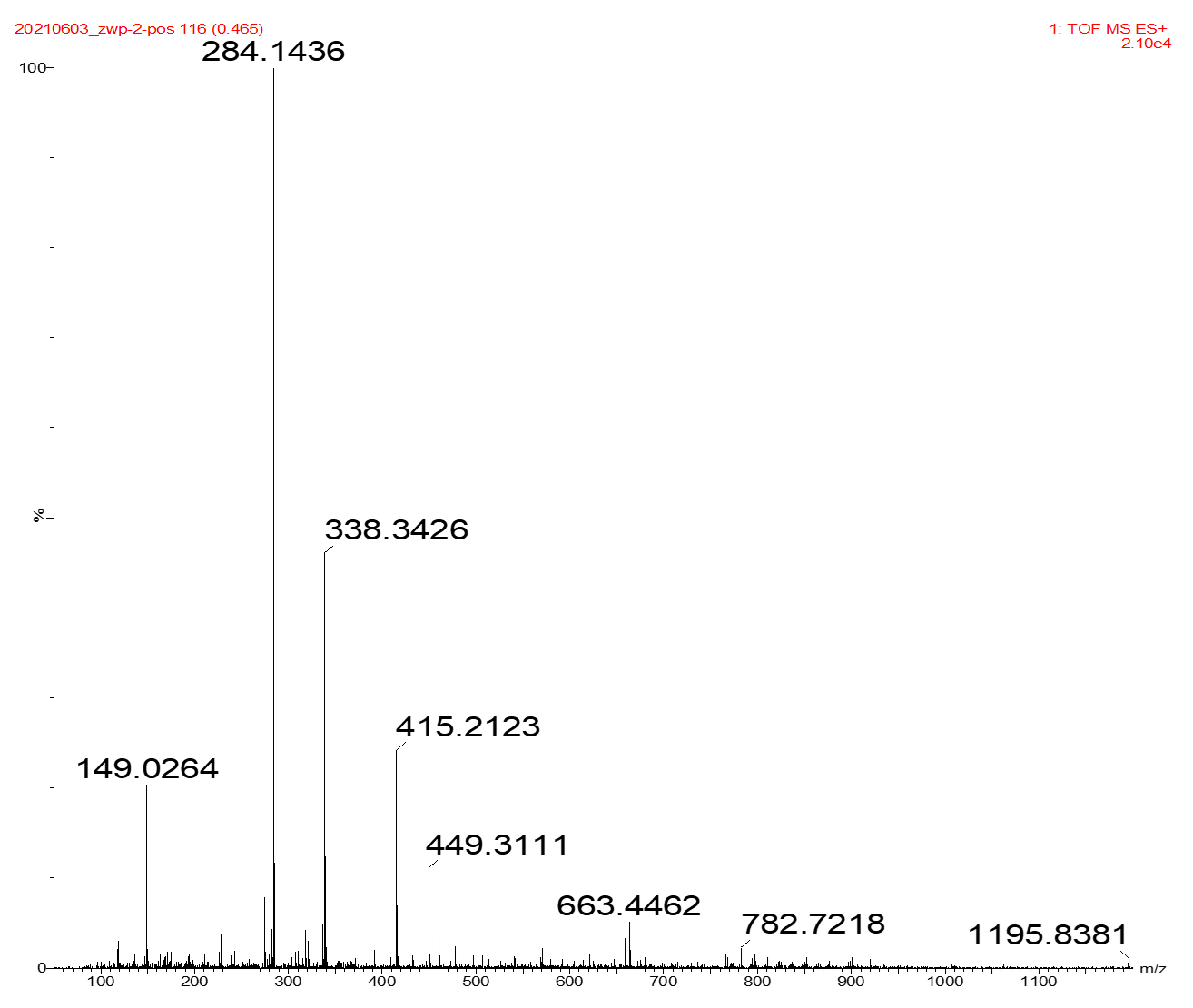


**Supplementary Figure 9.** HRMS spectra of (*Z*)-TPE-EPy.


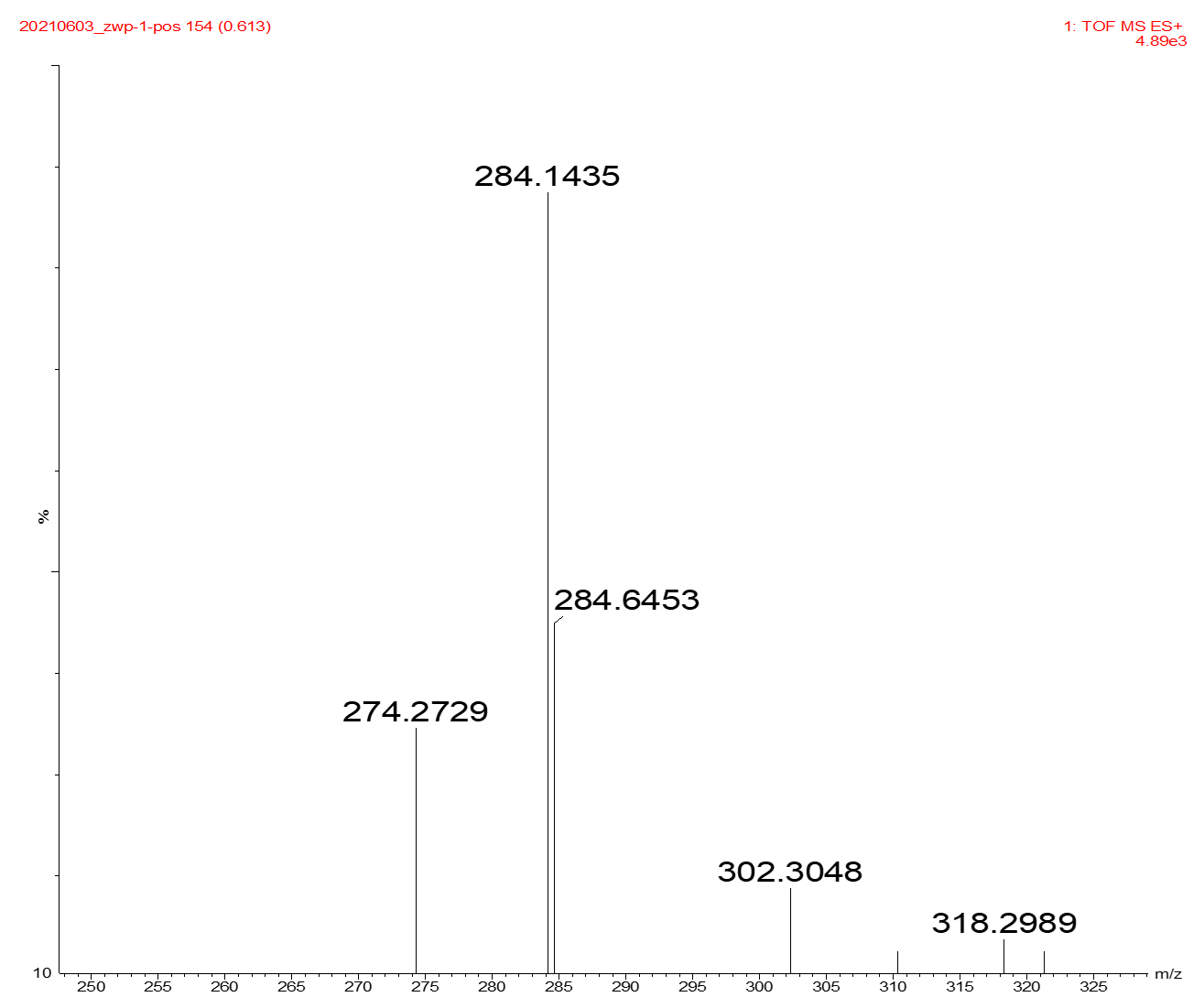


**Supplementary Figure 10.** HRMS spectra of (*E*)-TPE-EPy.

**Supplementary Table 1.** Crystallographic and structural refinement data of (*Z*)-TPE-EPy (CCDC 2165387) and (*E*)-TPE-EPy (CCDC 2165372). [a]

| Identification code | (*Z*)-TPE-EPy | (*E*)-TPE-EPy |
| --- | --- | --- |
| Empirical formula  Formula weight  Temperature / K  Crystal system  Space group  a / Å  b / Å  c / Å  α / °  β / °  γ /°  Volume / Å^3^  Z | C_42_H_36_I_2_N_2_  822.53  170.00(14)  Tetragonal  P4_2_/n  42.1139(3)  42.1139(3)  9.22650(10)  90  90  90  16363.9(3)  16 | C_42_H_36_I_2_N_2_  822.53  112.10(14)  Monoclinic  I_2_/a  17.058(2)  16.4766(18)  32.030(5)  90.00  103.272(16)  90.00  8762(2)  8 |

[a] Deposition Number 2165387 and 2165372 contains the supplementary crystallographic data for this paper. These data are provided free of charge by the joint Cambridge Crystallographic Data Centre and Fachinformationszentrum Karlsruhe Access Structures service www.ccdc.cam.ac.uk/structures.


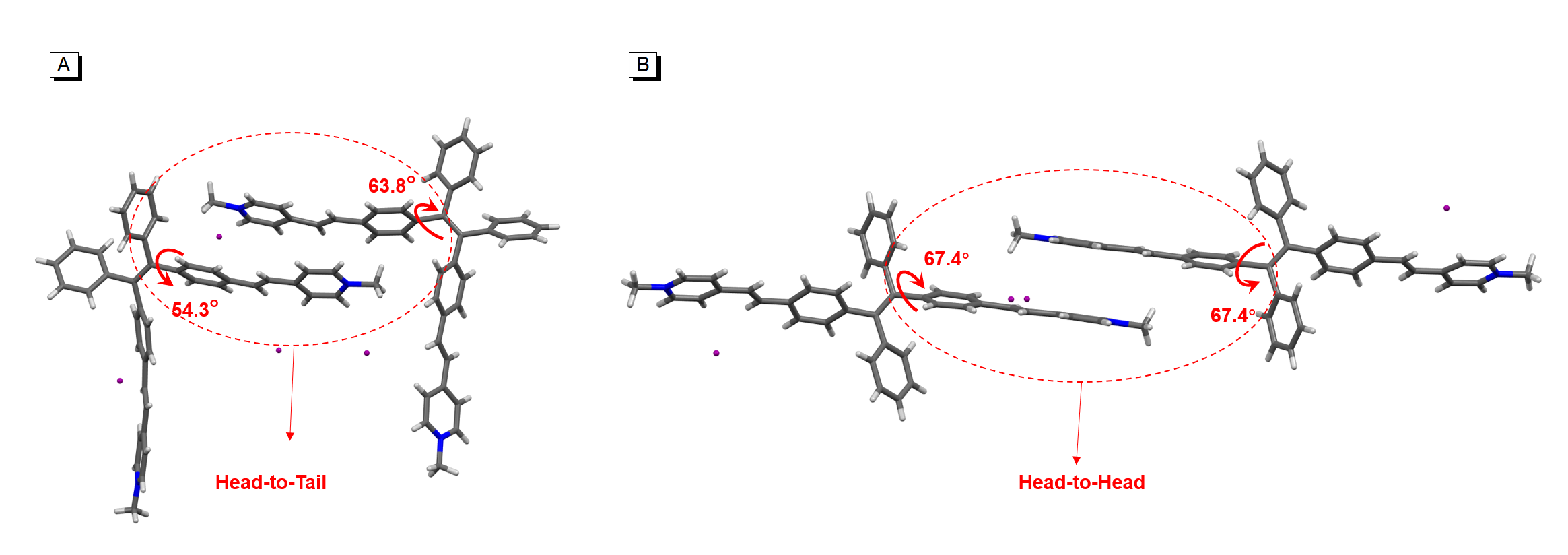


**Supplementary Figure 11.** Intermolecular stacking of (*Z*)-TPE-EPy and (*E*)-TPE-EPy in crystal.


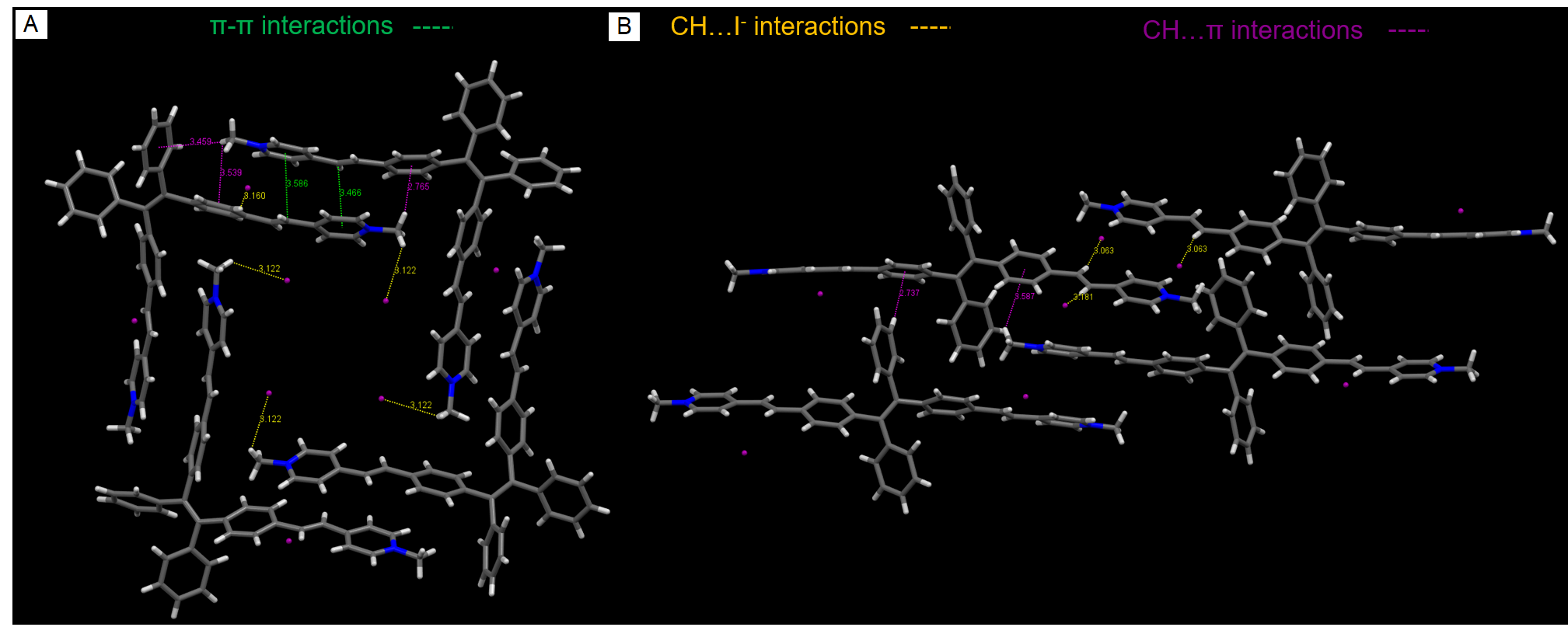


**Supplementary Figure 12.** Single-crystal X-ray diffraction analysis of (*Z*)-TPE-EPy and (*E*)-TPE-EPy.


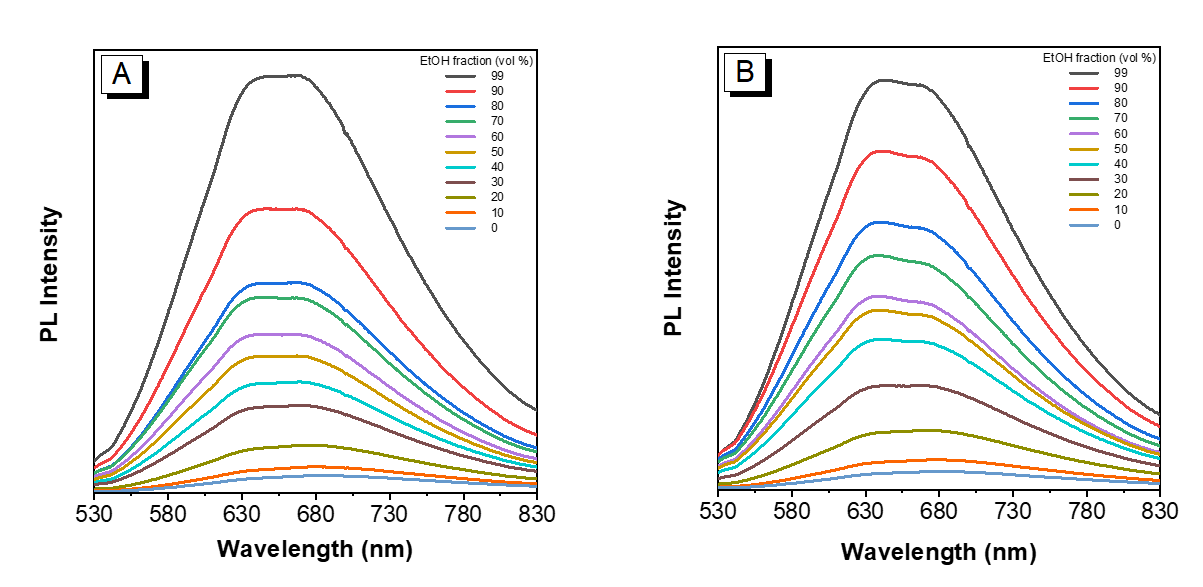


**Supplementary Figure 13.** A) (*Z*)-TPE-EPy and B) (*E*) -TPE-EPy (10 μM) in H_2_O/EtOH mixtures with different EtOH fractions (vol %).


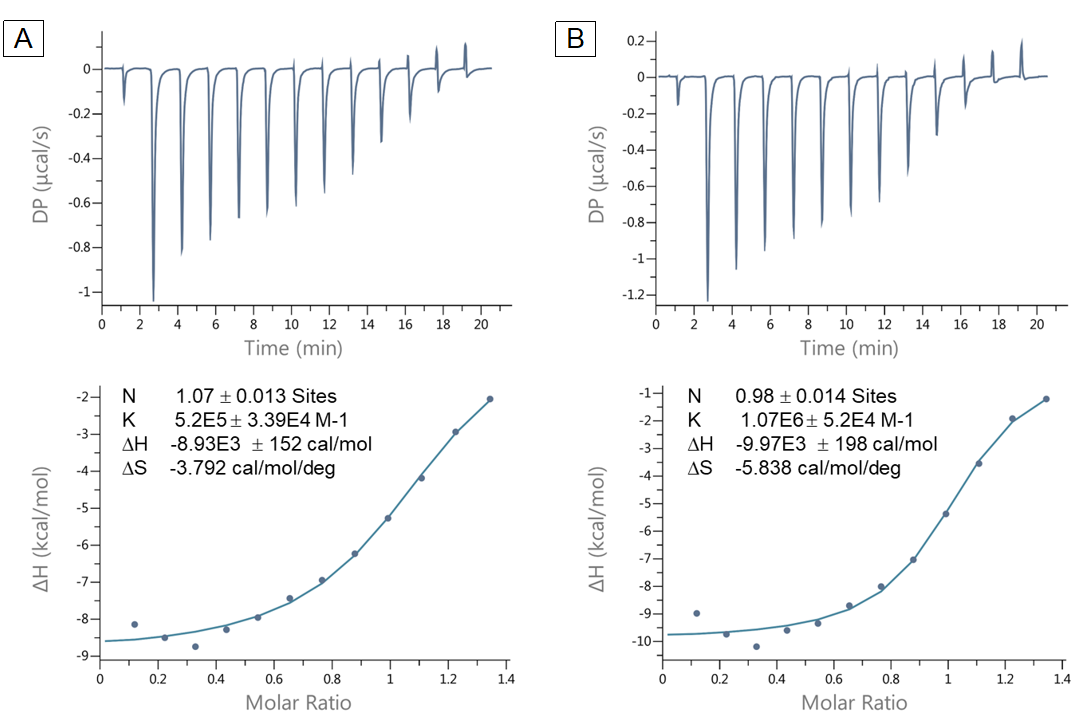


**Supplementary Figure 14.** ITC data for (*Z*)-TPE-EPy and (*E*)-TPE-EPy with CB[8] in H_2_O. [(*Z*)-TPE-EPy] = [(*E*)-TPE-EPy] (cell)=50 μM, [CB[8]] (syringe)=350 μM, 298 K.


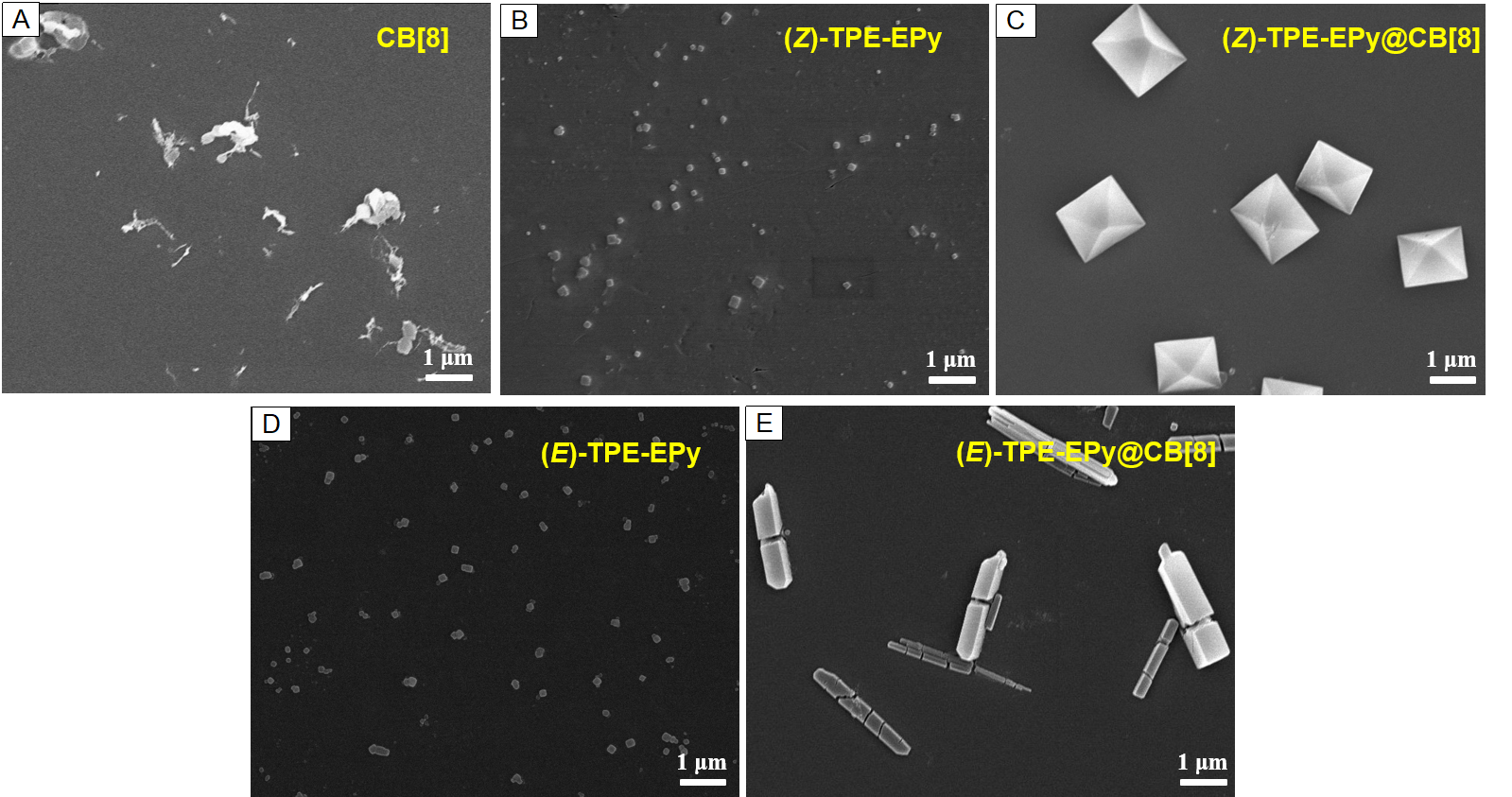


**Supplementary Figure 15.** SEM images of A) CB[8], B) (*Z*)-TPE-EPy, C) (*Z*)-TPE-EPy@CB[8], D) (*E*)-TPE-EPy and E) (*E*)-TPE-EPy@CB[8]. The concentration of each sample was 10 μM and the scale bar was 1 μm. Experiments were performed three times independently, representative images are shown.


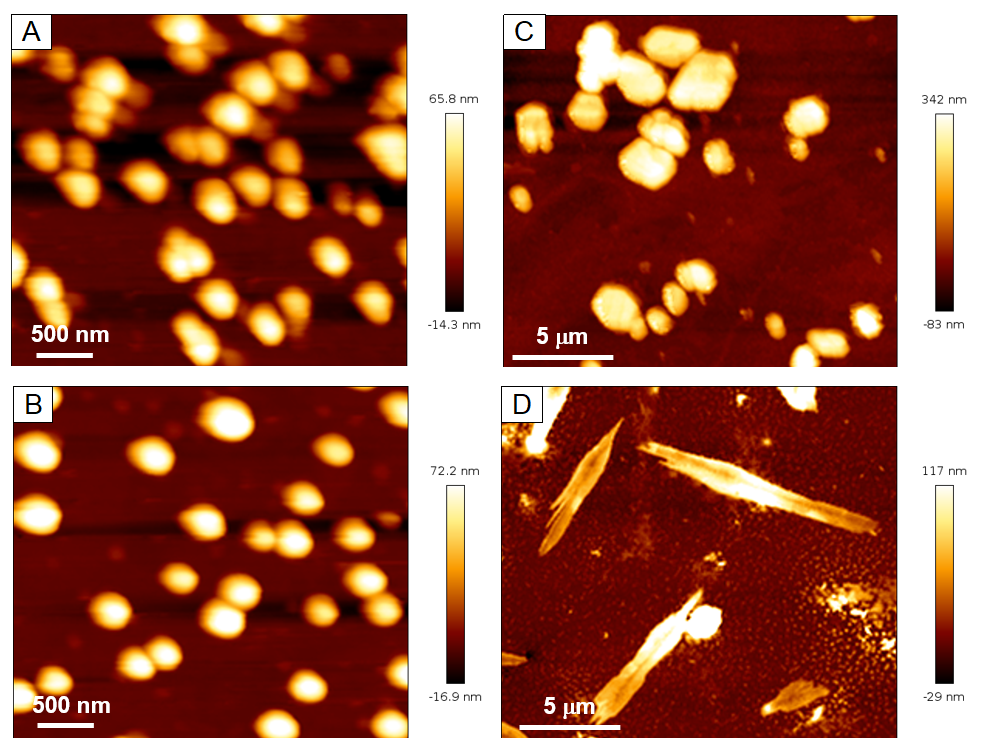


**Supplementary Figure 16.** AFM images of A) (*Z*)-TPE-EPy, B) (*E*)-TPE-EPy, C) (*Z*)-TPE-EPy@CB[8], D) (*E*)-TPE-EPy@CB[8]. The concentration of each sample was 10 μM.


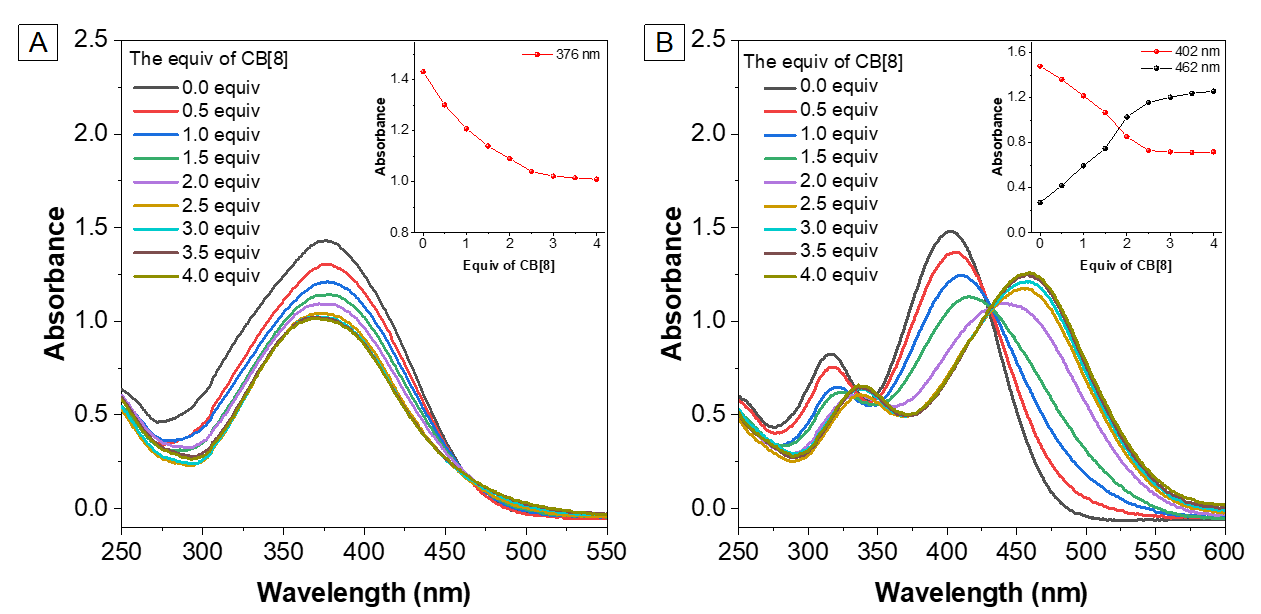


**Supplementary Figure 17.** A) UV-vis absorption titration spectra of (*Z*)-TPE-EPy (30 μM) with different amounts of CB[8] in ultrapure water. Insets: Plots of the absorbance at 376 nm. B) UV-vis absorption titration spectra of (*E*) -TPE-EPy (30 μM) with different amounts of CB[8] in ultrapure water. Insets: Plots of the absorbance intensity at 402 or 462 nm. The changes of the UV-vis spectra of (*Z*)-TPE-EPy were not obvious compared to those of the (*E*)-isomer with CB[8] titration, presumably as a result of the steric hindrance from the V-shaped (*Z*)-configuration, which hampered the host-enhanced π-π interaction of the end groups on two separate (*Z*)-TPE-EPy molecules.


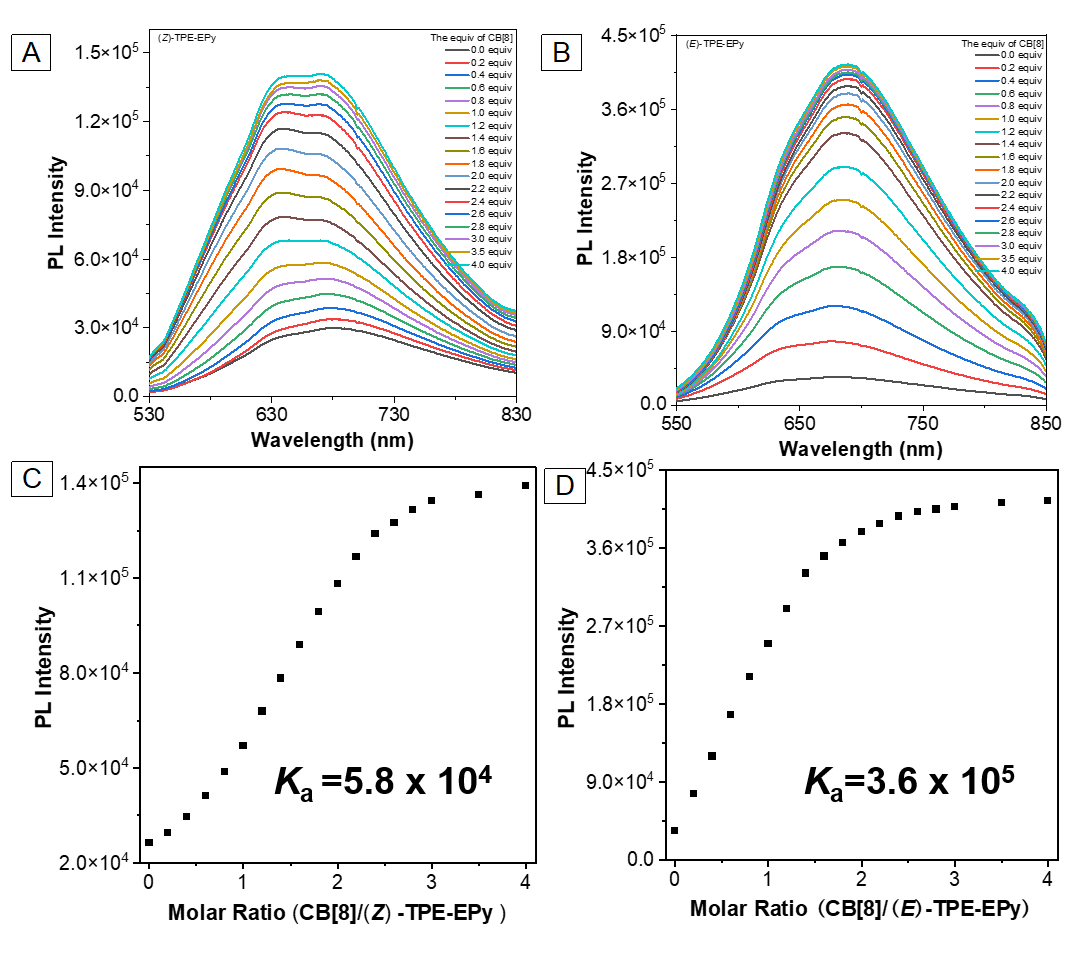


**Supplementary Figure 18.** A) Fluorescence titration spectra of (*Z*)-TPE-EPy (30 μM) with different amounts of CB[8] (from 0 to 4 equiv) in ultrapure water. B) Fluorescence titration spectra of (*E*) -TPE-EPy (30 μM) with different amounts of CB[8] (from 0 to 4 equiv) in ultrapure water. The linear fittingcurves of fluorescence intensity C) from Figure A) at 638 nm and D) from Figure B) at 686 nm.


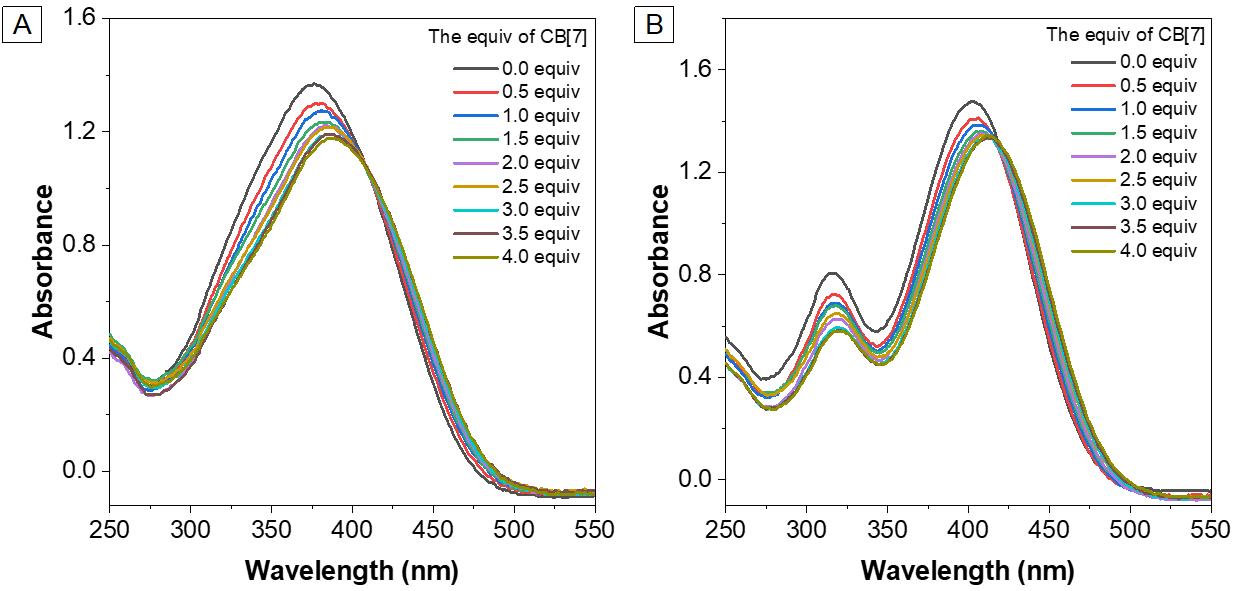


**Supplementary Figure 19.** A) UV-vis absorption titration spectra of (*Z*)-TPE-EPy (30 μM) with different amounts of CB[7] in ultrapure water. B) UV-vis absorption titration spectra of (*E*)-TPE-EPy with different amounts of CB[7] in ultrapure water.


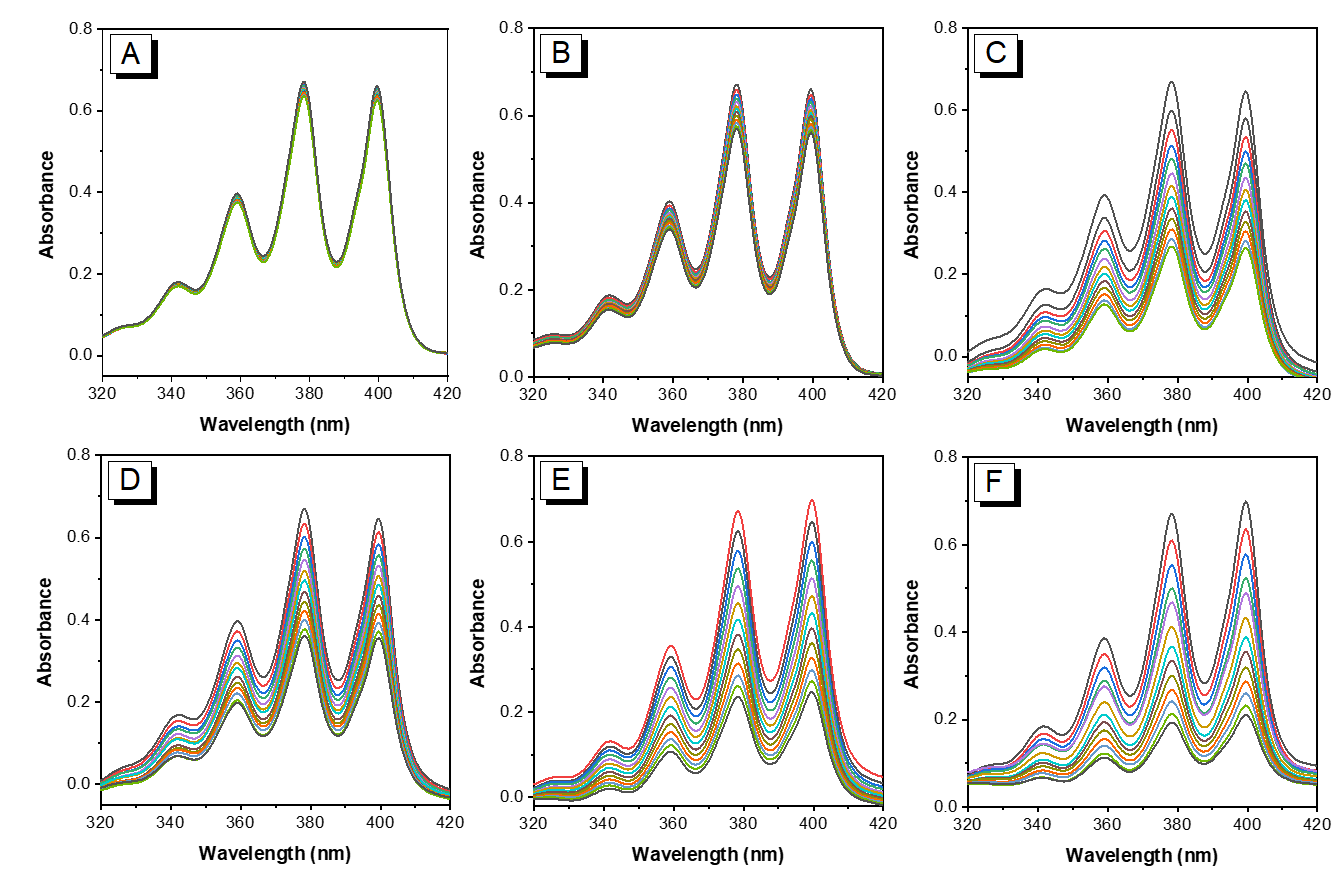


**Supplementary Figure 20.** A) UV-vis spectra of ABDA in the absence of PSs under white light irradiation (26 mW**·**cm^-2^) in aqueous solution. UV-vis spectra of ABDA in the presence of B) Rose Bengal, C) (*Z*)-TPE-EPy, D) (*Z*)-TPE-EPy@CB[8], E) (*E*)-TPE-EPy, and F) (*E*)-TPE-EPy@CB[8] under white light irradiation (26 mW**·**cm^-2^) in the aqueous solution. Concentrations of Rose Bengal and (*Z*)-TPE-EPy, (*E*)-TPE-EPy, (*Z*)-TPE-EPy@CB[8], (*E*)-TPE-EPy@CB[8] are 50 μM and 5 μM. Time interval for UV measurement: 10s. Time interval for UV measurement:10s.


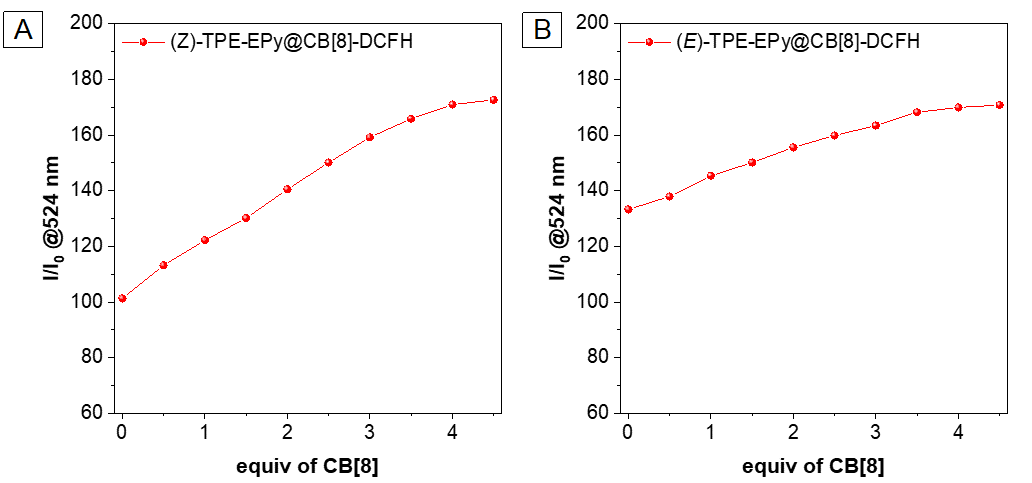


**Supplementary Figure 21.** ROS generation from A) (*Z*)-TPE-EPy and B) (*E*)-TPE-EPy with different equivalents of CB[8], upon white light irradiation for 2 minutes, using DCFH (5 μM) as an indicator.


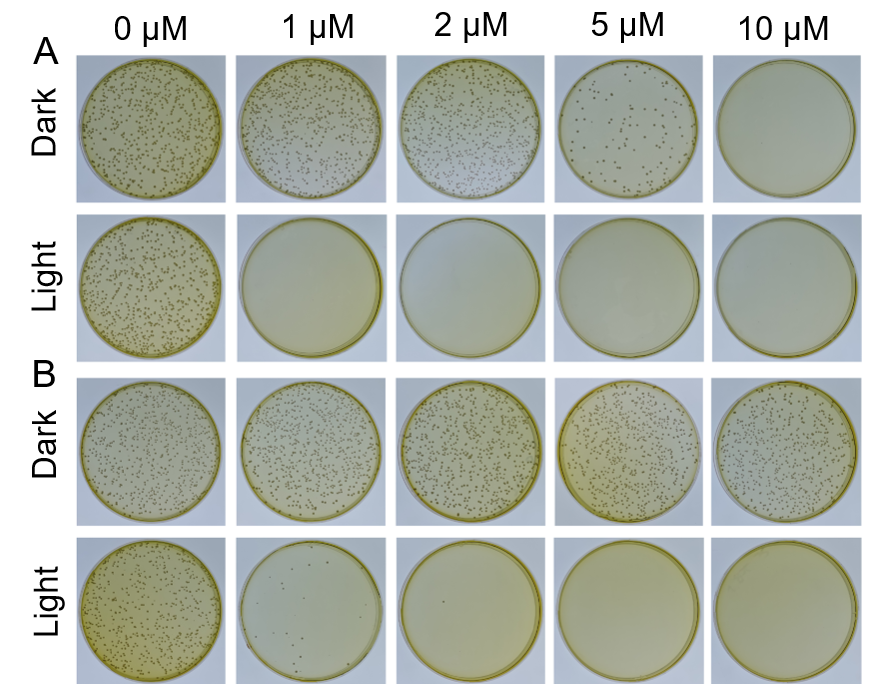


**Supplementary Figure 22.** Photographs of *S. cerevisiae* cultured on yeast agar plate supplemented with different concentration of A) (*Z*)-TPE-EPy, and B) (*Z*)-TPE-EPy@CB[8], in the darkness or upon white light irradiation (16 mW cm^-2^) for 10 min (n = 3).


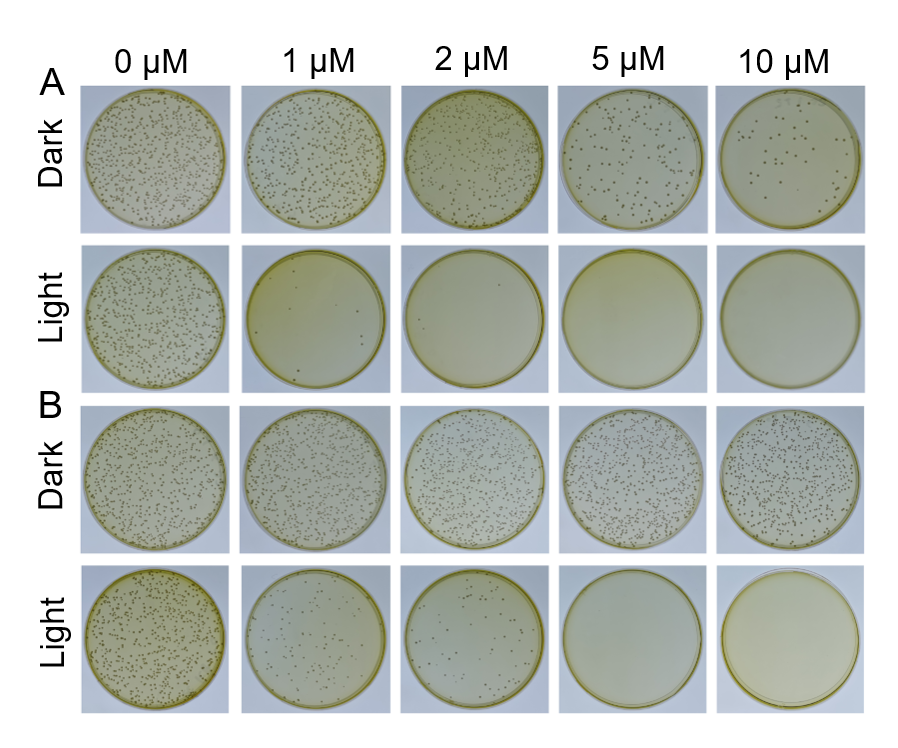


**Supplementary Figure 23.** Photographs of *S. cerevisiae* cultured on yeast agar plate supplemented with different concentration of A) (*E*)-TPE-EPy, and B) (*E*)-TPE-EPy@CB[8], in the darkness or upon white light irradiation (16 mW cm^-2^) for 10 min (n = 3).


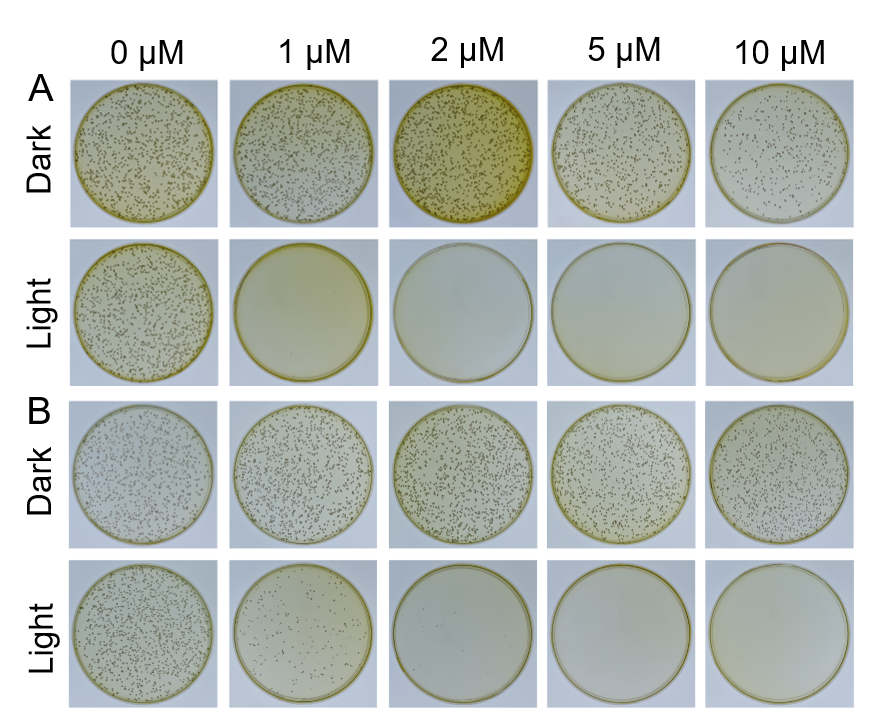


**Supplementary Figure 24.** Photographs of *C. albicans* cultured on yeast agar plate supplemented with different concentration of A) (*Z*)-TPE-EPy, and B) (*Z*)-TPE-EPy@CB[8], in the darkness or upon white light irradiation (16 mW cm^-2^) for 10 min (n = 3).


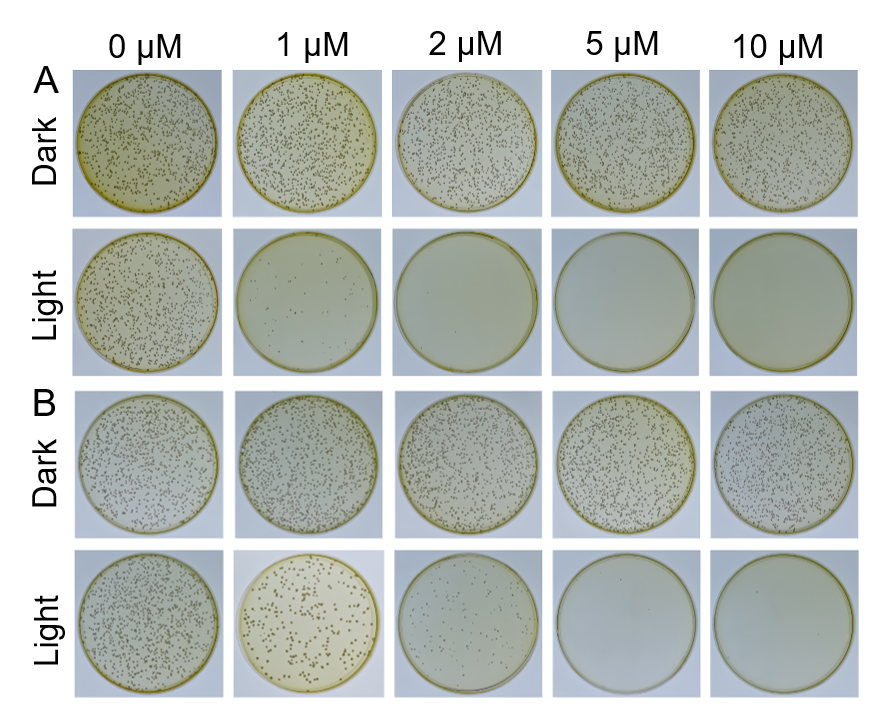


**Supplementary Figure 25.** Photographs of *C. albicans* cultured on agar plate supplemented with different concentration of A) (*E*)-TPE-EPy, and B) (*E*)-TPE-EPy@CB[8], in the darkness or upon white light irradiation (16 mW cm^-2^) for 10 min (n = 3).


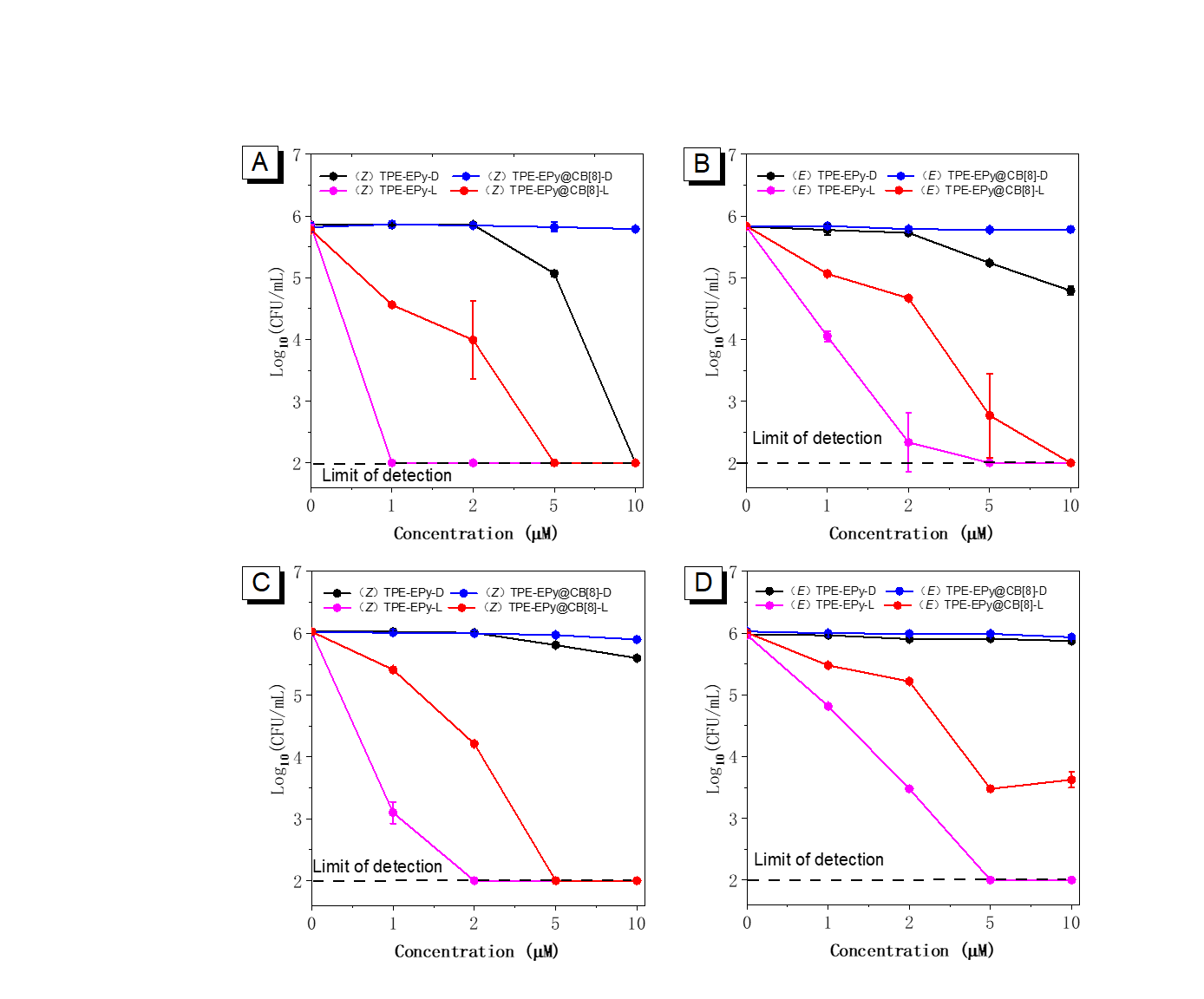


**Supplementary Figure 26.** Colony count of A), B) *S. cerevisiae* and C), D) *C. albicans* in darkness or unpon light irradiation (16 mW cm^-2^) following the treatment of different concentrations of (*Z*)-TPE-EPy, (*E*)-TPE-EPy, (*Z*)-TPE-EPy@CB[8], (*E*)-TPE-EPy@CB[8] for 10 min, respectively. Dashed lines indicate limit of detection. Data are presented as mean _± SD (n = 3 biological independent samples).


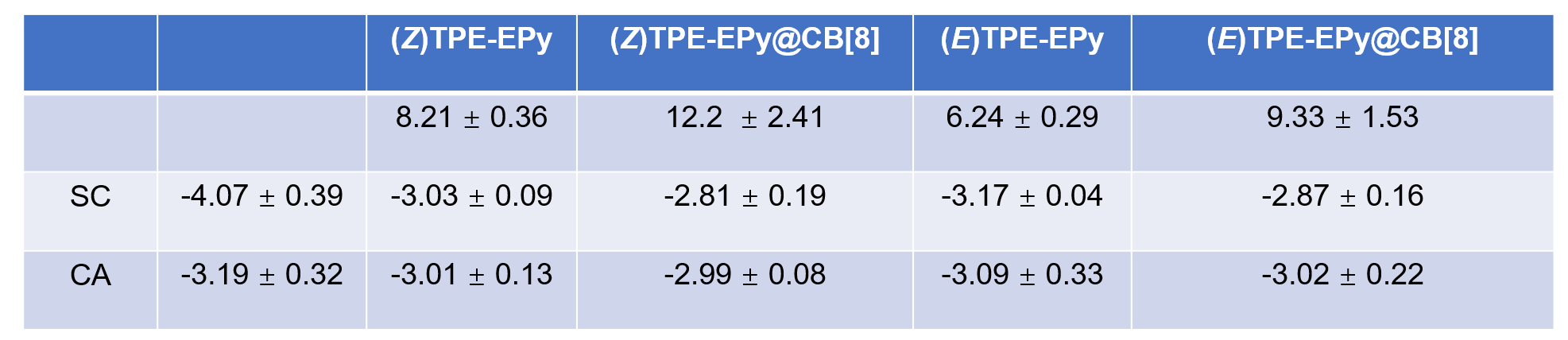


**Supplementary Figure 27.** Zeta potential results of *S. cerevisiae* (SC) and *C. albicans* (CA) in PBS solution pre-treated with or without (*Z*)-TPE-EPy, (*E*)-TPE-EPy, (*Z*)-TPE-EPy@CB[8], and (*E*)-TPE-EPy@CB[8].


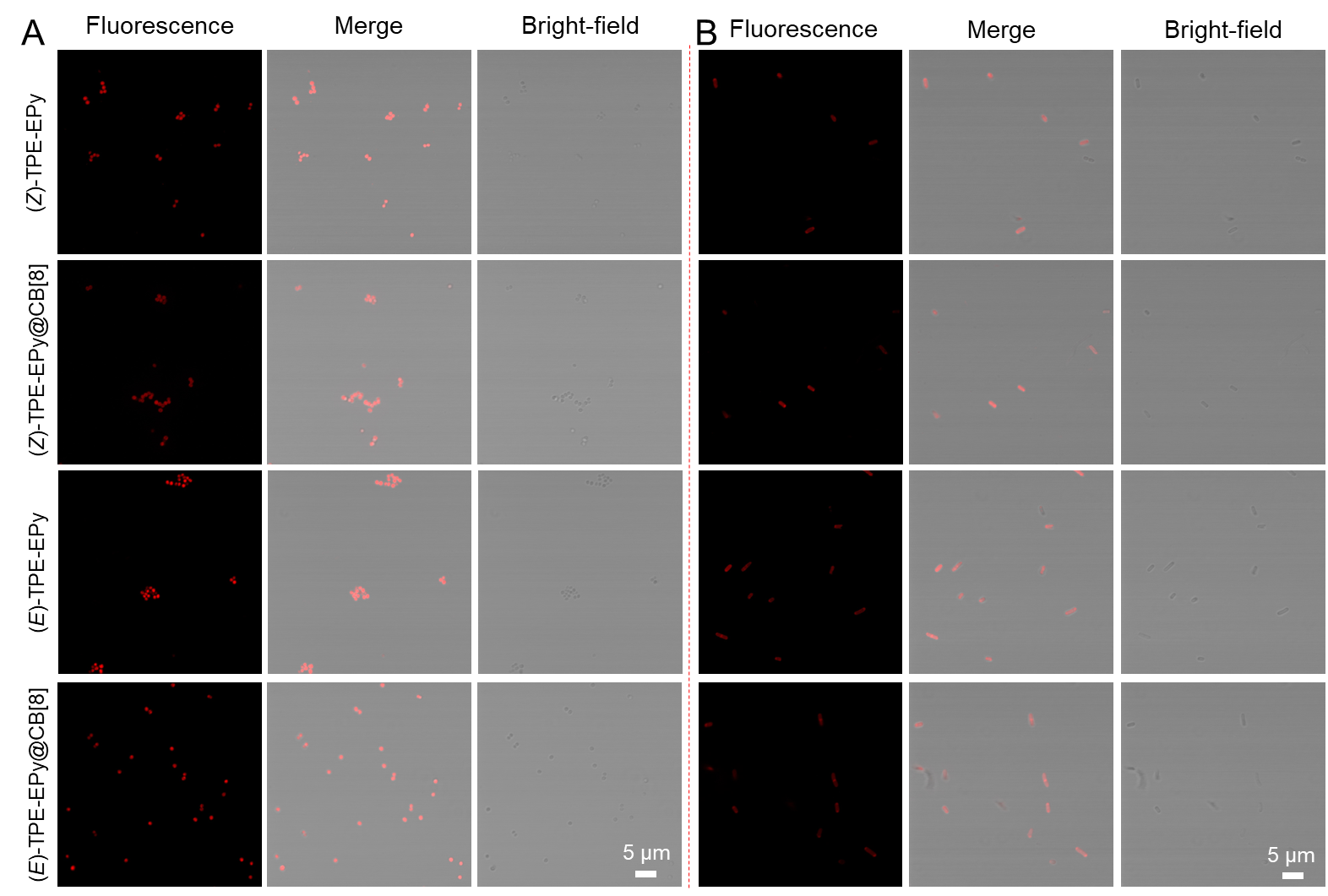


**Supplementary Figure 28.** Bright-field and fluorescent images of *S. aureus*  A) and *E.coli* B) incubated with the different AIEgen (5 µM) for 10 min. (*Z*)-TPE-EPy, (*E*)-TPE-EPy, (*Z*)-TPE-EPy@CB[8]: λ_ex_ = 405 nm, λ_em_ = 600-700. Experiments were performed three times independently, representative images are shown.


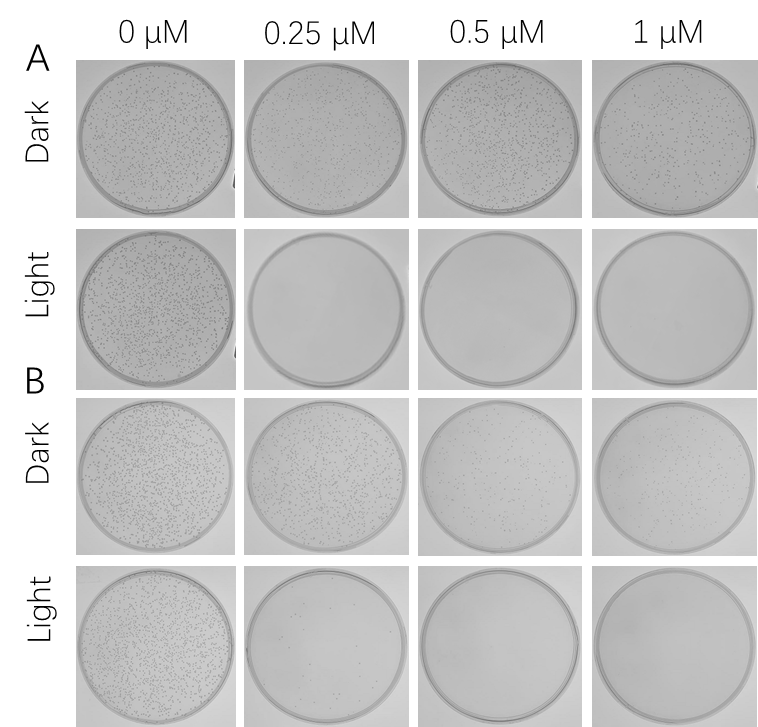


**Supplementary Figure 29.** Photographs of *S. aureus* cultured on agar plate supplemented with different concentration of (*Z*)-TPE-EPy (A) and (*Z*)-TPE-EPy@CB[8] (B) in darkness or upon white light irradiation (16 mW cm^-2^) for 10 min (n=3)


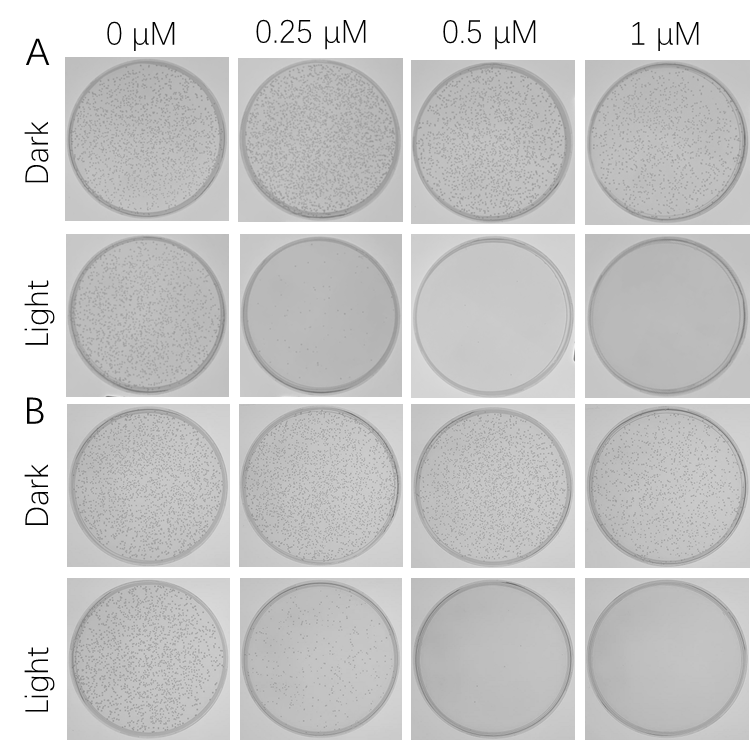


**Supplementary Figure 30.** Photographs of *S. aureus* cultured on agar plate supplemented with different concentration of (*E*)-TPE-EPy (A) and (*E*)-TPE-EPy@CB[8] (B) in darkness or upon white light irradiation (16 mW cm^-2^) for 10 min (n=3)


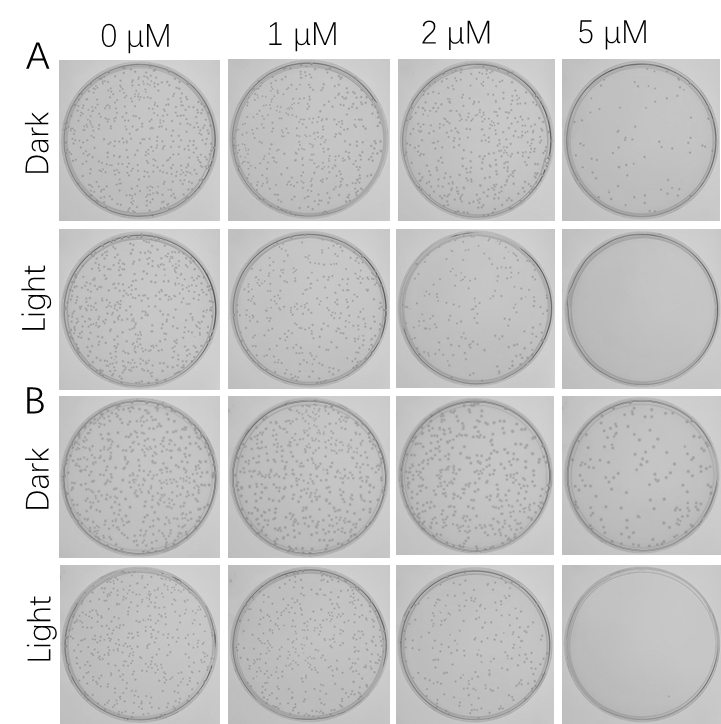


**Supplementary Figure 31.** Photographs of *E.coli* cultured on agar plate supplemented with different concentration of (*Z*)-TPE-EPy (A) and (*Z*)-TPE-EPy@CB[8] (B) in the darkness or upon white light irradiation (16 mW cm^-2^) for 10 min (n=3)


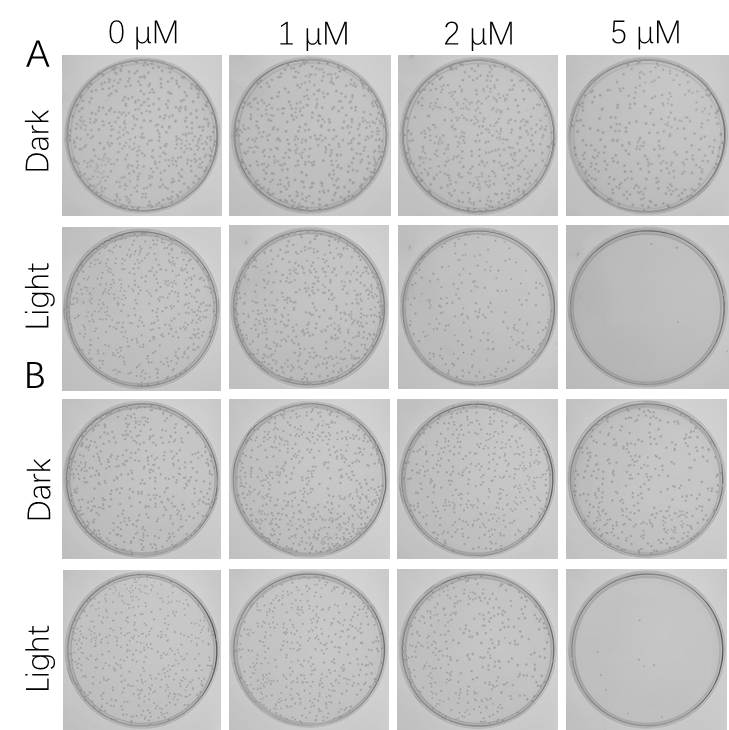


**Supplementary Figure 32.** Photographs of *E.coli* cultured on agar plate supplemented with different concentration of (*E*)-TPE-EPy (A) and (*E*)-TPE-EPy@CB[8] (B) in the darkness or upon white light irradiation (16 mW cm^-2^) for 10 min (n=3)


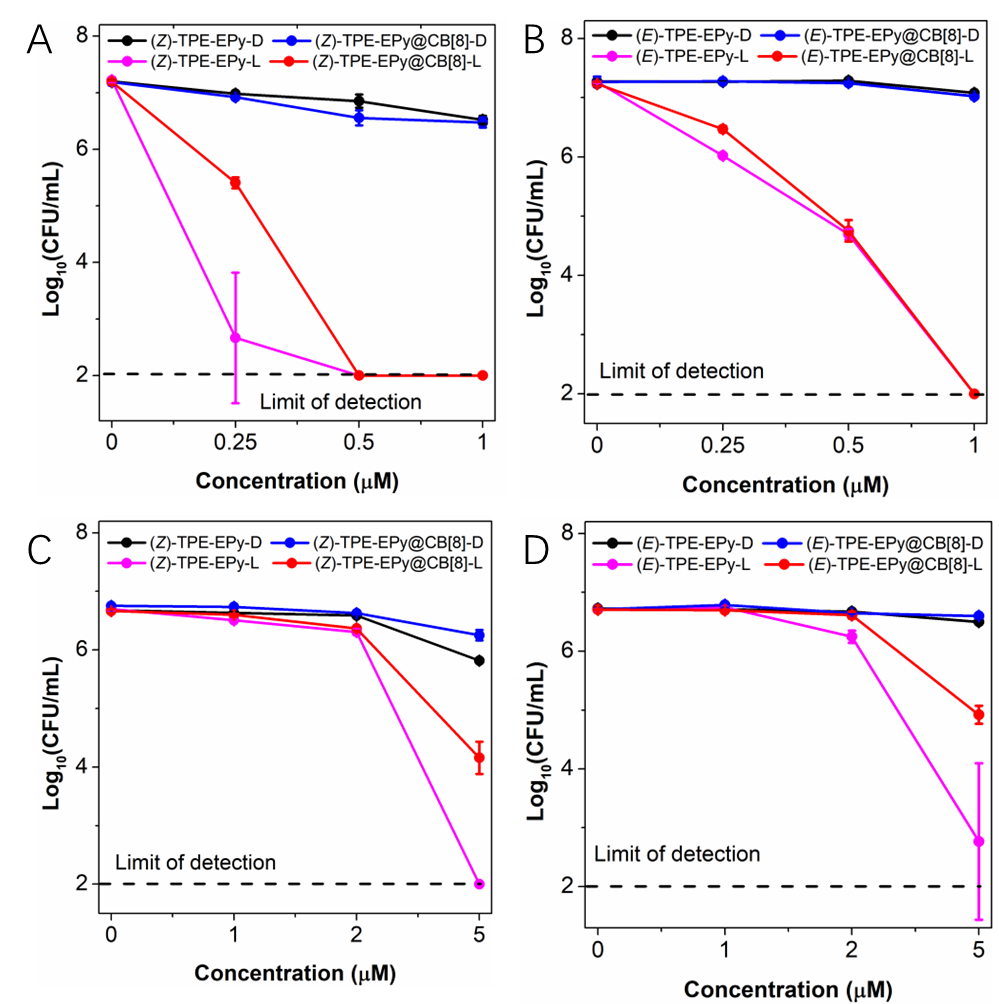


**Supplementary Figure 33.** Colony count of A), B) *S. aureus* and C), D) *E.coli* in the darkness or unpon light irradiation (16 mW cm^-2^) following the treatment of different concentrations of (*Z*)-TPE-EPy, (*E*)-TPE-EPy, (*Z*)-TPE-EPy@CB[8], (*E*)-TPE-EPy@CB[8] for 10 min, respectively. Dashed lines indicate limit of detection. Data are presented as mean _± SD (n = 3 biological independent samples).


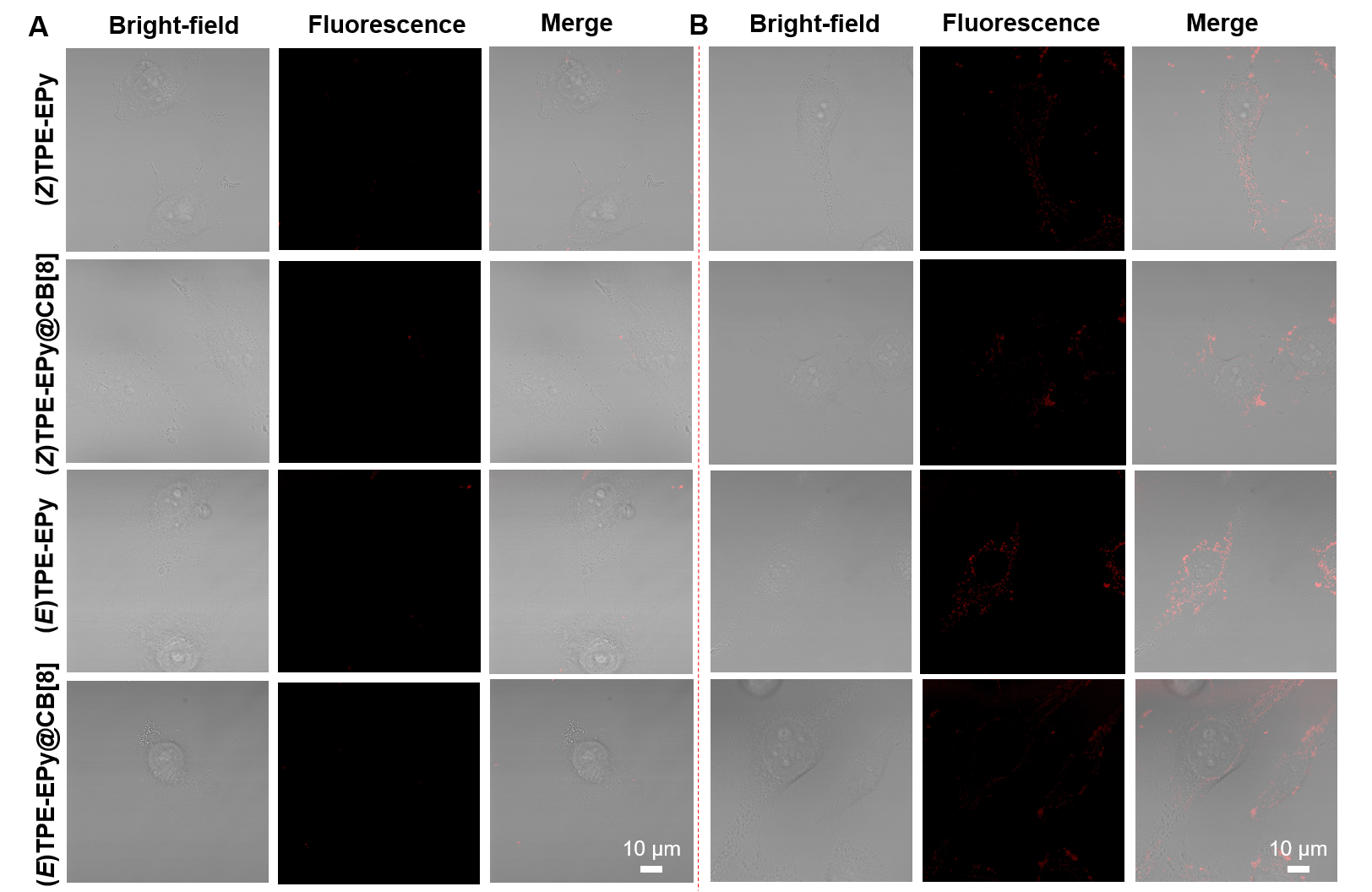


**Supplementary Figure 34.** Bright-field and fluorescent images of HUVECs incubated with A) 2 μM and B) 10 μM of the stereoisomers for 10 min. (*Z*)-TPE-EPy, (*E*)-TPE-EPy, (*Z*)-TPE-EPy@CB[8]: λ_ex_ = 405 nm, λ_em_ = 600-640 nm (*E*)-TPE-EPy@CB[8]: λ_ex_ = 488 nm, λ_em_ = 600-640 nm. Experiments were performed three times independently, representative images are shown.


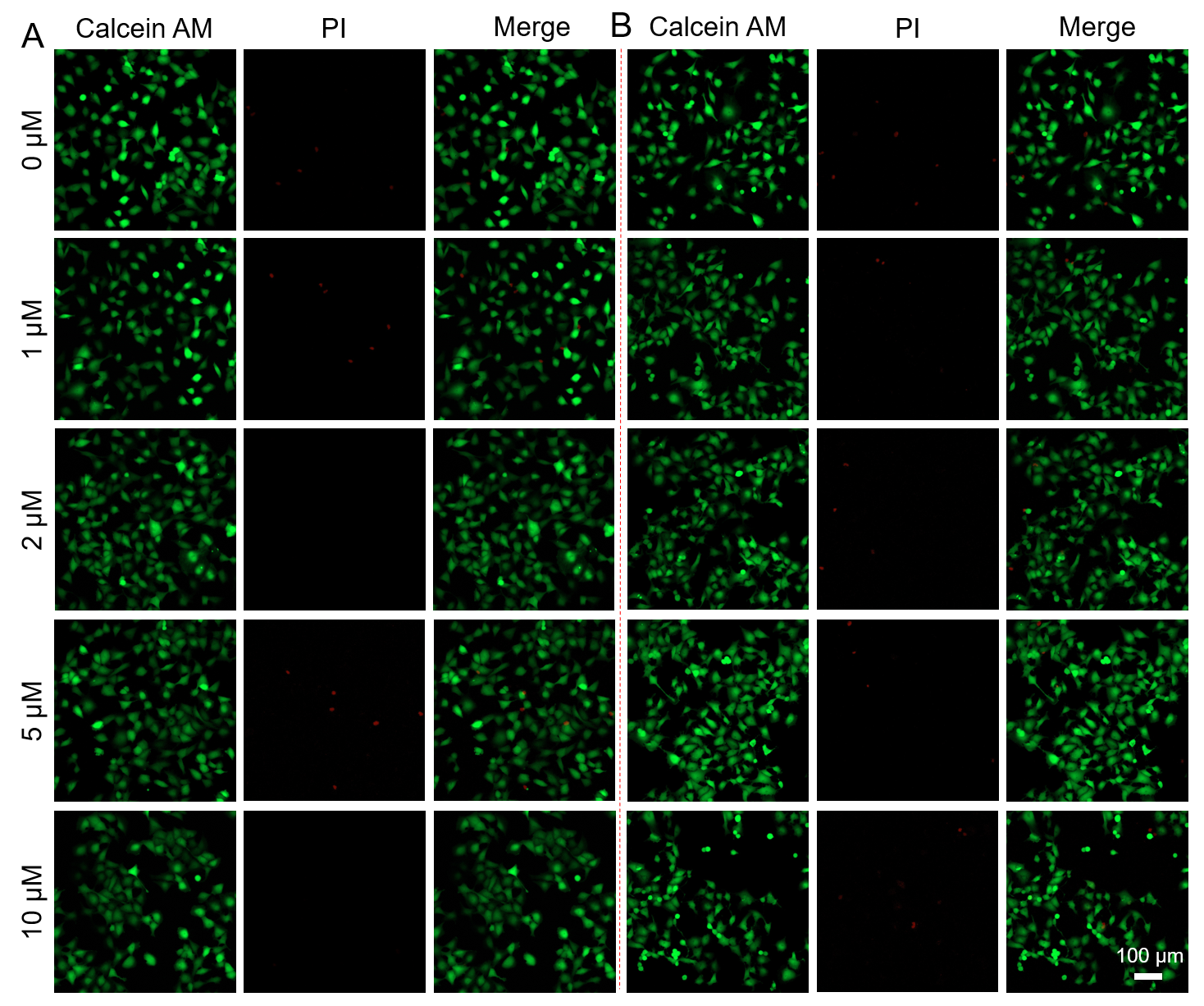


**Supplementary Figure 35.** Fluorescence images of calcein AM/PI stained HUVECs and cells treated by different concentrations of A) (*Z*)-TPE-EPy in darkness and B) upon light irradiation (16 mW**·**cm^-2^) for 10 min. The fields of vision were randomly selected. Calcein AM: λ_ex_ = 488 nm, λ_em_= 500-550 nm; PI: λ_ex_= 561 nm, λ_em_= 600-700 nm. Experiments were performed three times independently, representative images are shown.


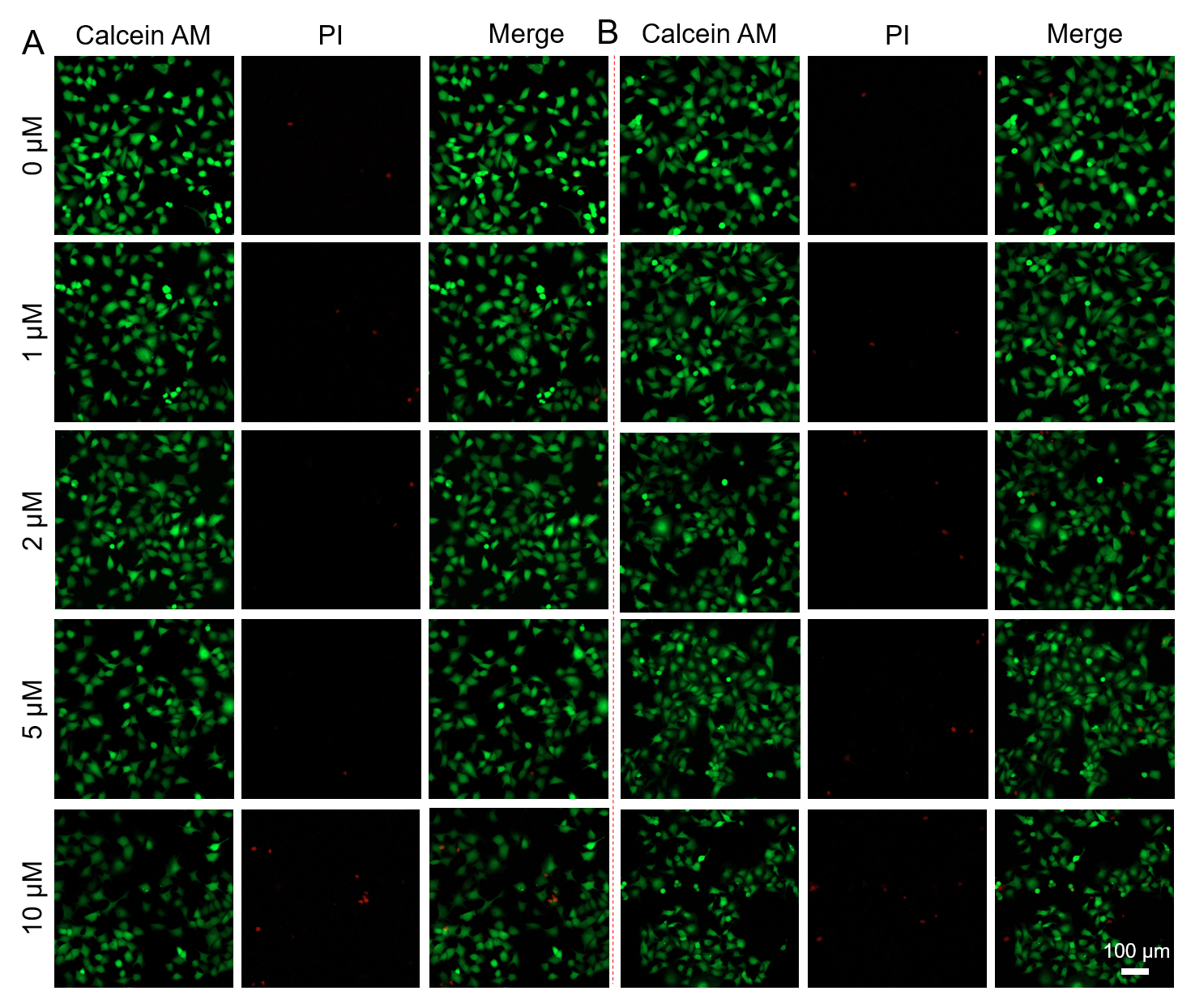


**Supplementary Figure 36.** Fluorescence images of calcein AM/PI stained HUVECs and cells treated by different concentrations of A) (*Z*)-TPE-EPy@CB[8] in darkness and B) upon light irradiation (16 mW**·**cm^-2^) for 10 min. The fields of vision were randomly selected. Calcein AM: λ_ex_ = 488 nm, λ_em_ = 500-550 nm; PI: λ_ex_ = 561 nm, λ_em_ = 600-700 nm. Experiments were performed three times independently, representative images are shown.


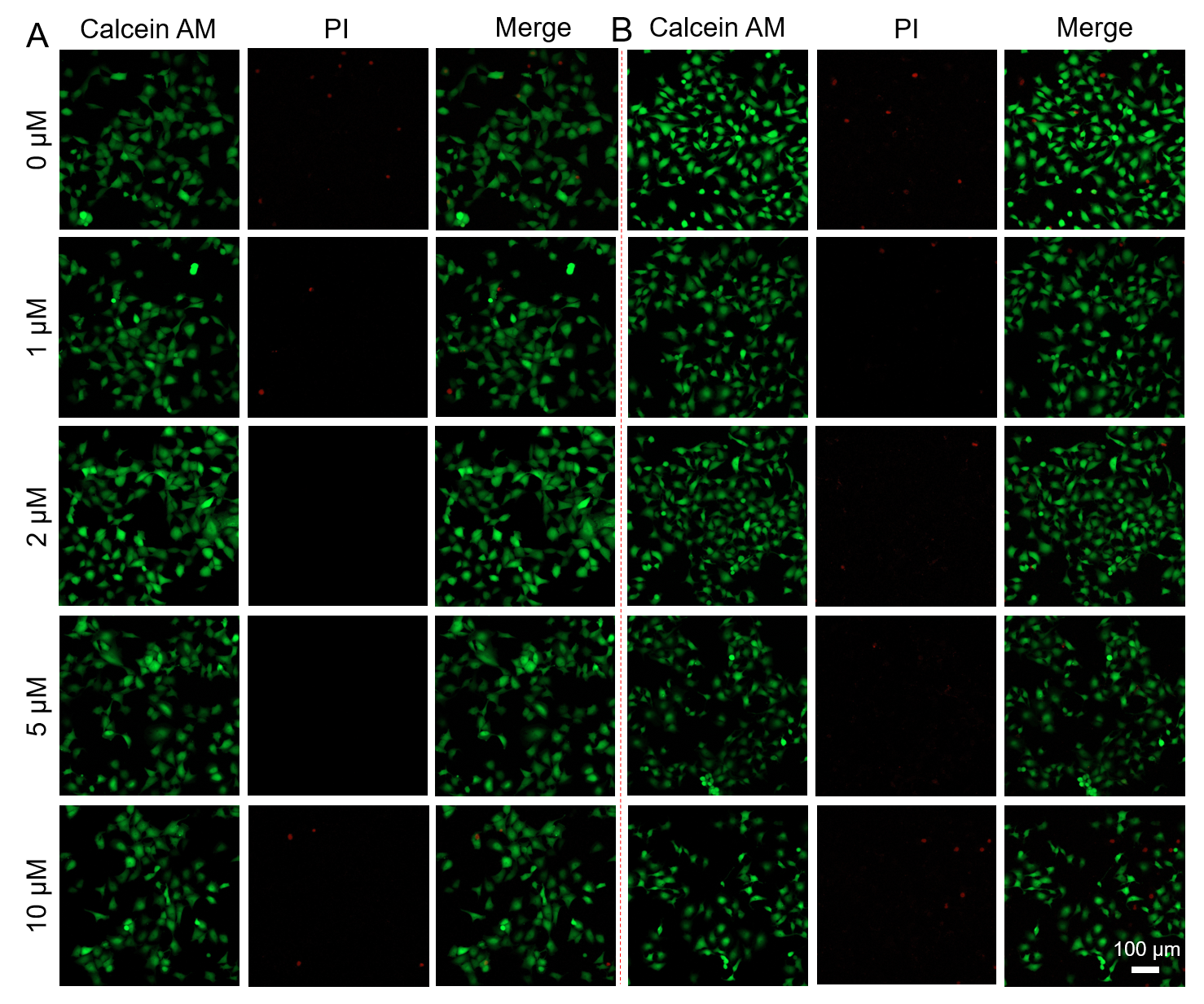


**Supplementary Figure 37.** Fluorescence images of calcein AM/PI stained HUVECs and cells treated by different concentrations of A) (*E*)-TPE-EPy in darkness and B) upon light irradiation (16 mW**·**cm^-2^) for 10 min. The fields of vision were randomly selected. Calcein AM: λ_ex_ = 488 nm, λ_em_ = 500-550 nm; PI: λ_ex_ = 561 nm, λ_em_ = 600-700 nm. Experiments were performed three times independently, representative images are shown.


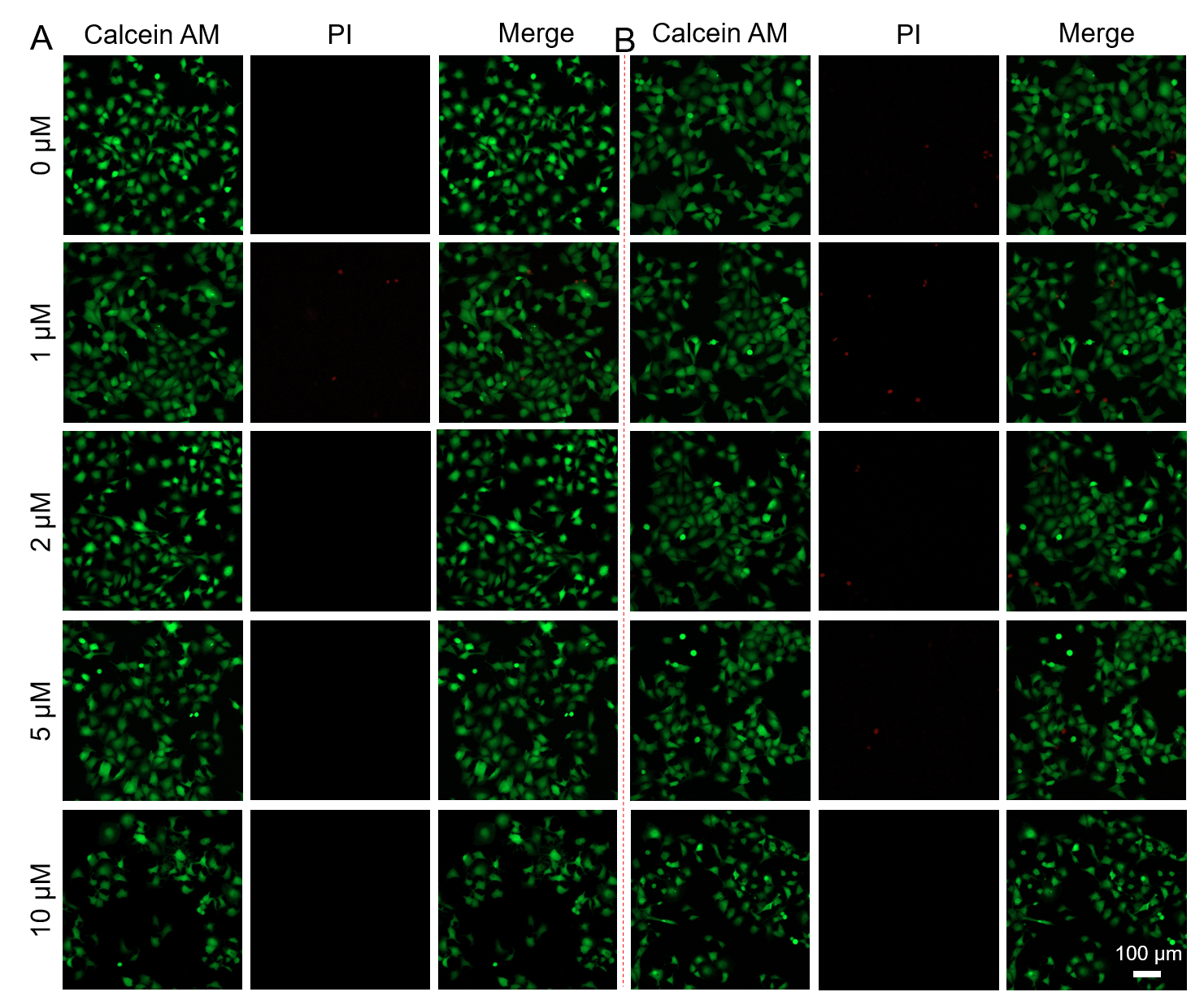


**Supplementary Figure 38.** Fluorescence images of calcein AM/PI stained HUVECs and cells treated by different concentrations of A) (*E*)-TPE-EPy@CB[8] in darkness and B) upon light irradiation (16 mW**·**cm^-2^) for 10 min. The fields of vision were randomly selected. Calcein AM: λ_ex_ = 488 nm, λ_em_ = 500-550 nm; PI: λ_ex_ = 561 nm, λ_em_ = 600-700 nm. Experiments were performed three times independently, representative images are shown.


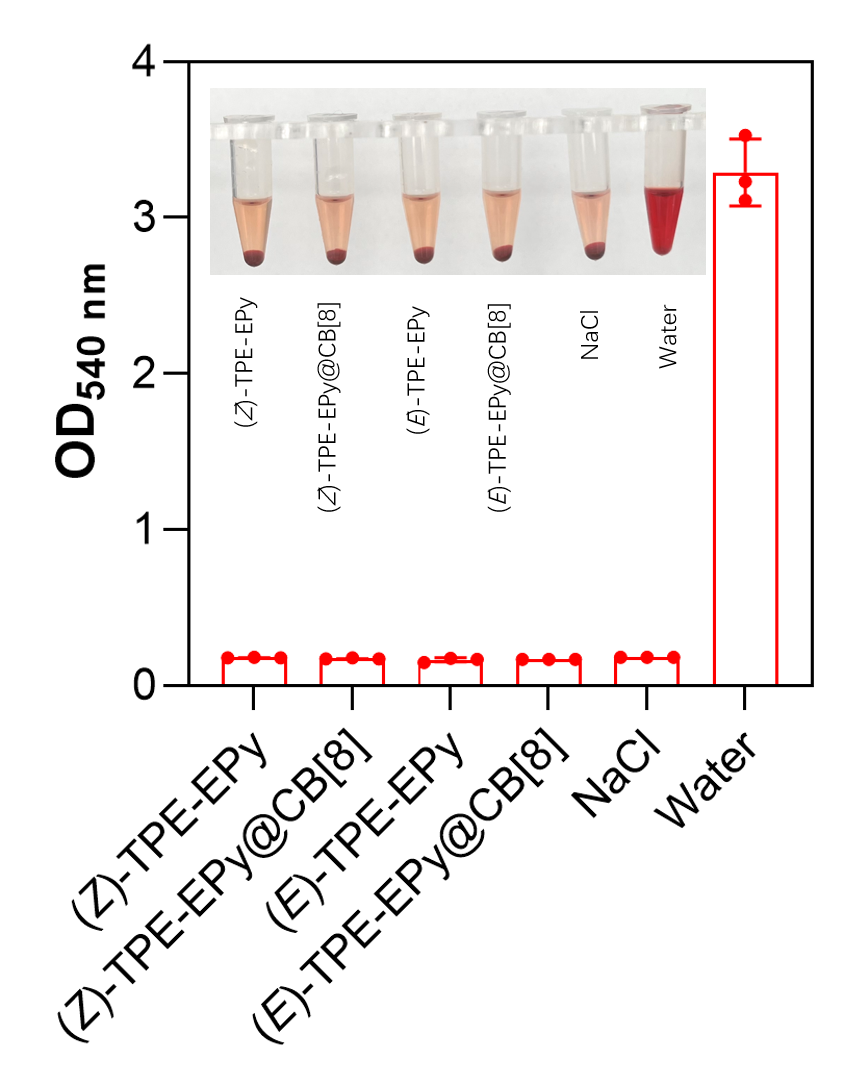


**Supplementary Figure 39.** Hemolysis activity of different AIE PSs (10 µM), NaCl (0.9% in water) and water. The results are presented as mean_± SD (n = 3 biological independent samples).

**Cartesian coordinates**

(*Z*)-TPE-EPy S_0_-geometry：

C -0.64964600 2.91781100 -0.19400900

C 0.64958200 2.91782200 0.19390400

C -1.47614600 1.68573900 -0.30110000

C -1.51567800 0.73494100 0.73101700

C -2.32161100 1.50681500 -1.40400400

C -2.38857800 -0.33766900 0.67908500

H -0.87385100 0.86446400 1.59637800

C -3.17928100 0.42229800 -1.46847600

H -2.31581600 2.24065100 -2.20361600

C -3.25192100 -0.50559200 -0.41688000

H -2.40414500 -1.04162500 1.50553900

H -3.83034900 0.30845200 -2.33144100

C 1.47610600 1.68576800 0.30100700

C 2.32153800 1.50685200 1.40393800

C 1.51570400 0.73498700 -0.73112300

C 3.17923500 0.42235800 1.46842600

H 2.31569500 2.24067800 2.20355900

C 2.38863100 -0.33760000 -0.67917400

H 0.87390900 0.86450700 -1.59650800

C 3.25193700 -0.50551600 0.41682100

H 3.83027500 0.30851800 2.33141300

H 2.40424800 -1.04154400 -1.50563800

C -1.37850400 4.17617900 -0.52654400

C -0.84868600 5.08675900 -1.44639300

C -2.62267100 4.44629900 0.05406500

C -1.53943600 6.25007300 -1.76438400

H 0.11369800 4.88386800 -1.90606500

C -3.30744700 5.61590100 -0.25517200

H -3.04958600 3.74083900 0.76228300

C -2.76723300 6.52041600 -1.16579700

H -1.11687100 6.94814200 -2.48009300

H -4.26398900 5.82204400 0.21498100

H -3.30293100 7.43221800 -1.41025600

C 1.37838700 4.17619700 0.52652500

C 0.84847200 5.08674400 1.44634800

C 2.62259100 4.44636300 -0.05398500

C 1.53916400 6.25007100 1.76441500

H -0.11394800 4.88382000 1.90593300

C 3.30731000 5.61597900 0.25532800

H 3.04957900 3.74092900 -0.76218500

C 2.76700000 6.52046000 1.16593000

H 1.11652100 6.94811700 2.48010200

H 4.26388300 5.82215800 -0.21474700

H 3.30265300 7.43227200 1.41044800

C 4.25762200 -1.55192600 0.49507200

H 4.76529400 -1.60772800 1.45706700

C 4.64573400 -2.38979800 -0.49348800

H 4.18449200 -2.31713700 -1.47441700

C -4.25758300 -1.55202500 -0.49510700

H -4.76529000 -1.60782800 -1.45708400

C -4.64563800 -2.38991700 0.49345900

H -4.18436300 -2.31725600 1.47437100

C 5.70660500 -3.36560300 -0.37941400

C 6.12071200 -4.07921500 -1.52496700

C 6.39443800 -3.66825700 0.81742000

C 7.14264100 -4.99164700 -1.46033800

H 5.63988700 -3.90896600 -2.48155900

C 7.40911300 -4.58908600 0.82762900

H 6.14042300 -3.18992800 1.75492700

H 7.48432300 -5.54292300 -2.32809200

H 7.95722400 -4.83575200 1.72912700

C -5.70649100 -3.36574600 0.37941400

C -6.12053900 -4.07938000 1.52497300

C -6.39436300 -3.66840400 -0.81739900

C -7.14245100 -4.99183500 1.46037400

H -5.63968300 -3.90913000 2.48155000

C -7.40901500 -4.58925500 -0.82757800

H -6.14039400 -3.19005700 -1.75491000

H -7.48408500 -5.54312600 2.32813700

H -7.95715700 -4.83592800 -1.72905700

N -7.78263800 -5.24527600 0.29653700

N 7.78279200 -5.24508700 -0.29647900

C 8.91500100 -6.18672100 -0.25605300

H 8.90797500 -6.71650000 0.69661700

H 8.80944200 -6.91003100 -1.06409000

H 9.85179200 -5.63731900 -0.37066000

C -8.91482100 -6.18694300 0.25611100

H -8.90754700 -6.71700800 -0.69640000

H -8.80945300 -6.91000600 1.06439300

H -9.85164800 -5.63752200 0.37032600

(*Z*)-TPE-EPy T_1_-geometry：

C -0.47724400 2.40810300 -0.57392700

C 0.47724300 2.40810300 0.57392900

C -1.52141700 1.44558500 -0.61545600

C -1.85202000 0.68152100 0.54479600

C -2.26851100 1.17295300 -1.79751800

C -2.90002000 -0.20330300 0.54922100

H -1.28179900 0.83492800 1.45551800

C -3.30327200 0.26870300 -1.78979800

H -2.00023400 1.66686800 -2.72412200

C -3.67502400 -0.42490300 -0.61542900

H -3.12898000 -0.73417600 1.46772400

H -3.85131400 0.08397500 -2.71020900

C 1.52141200 1.44558100 0.61546100

C 2.26852800 1.17297700 1.79751600

C 1.85199500 0.68149400 -0.54478100

C 3.30328500 0.26872300 1.78979900

H 2.00027100 1.66692000 2.72411200

C 2.89999200 -0.20333400 -0.54920400

H 1.28176000 0.83488400 -1.45549700

C 3.67501400 -0.42491100 0.61543900

H 3.85134400 0.08401600 2.71020300

H 3.12893500 -0.73422700 -1.46769900

C -0.29979600 3.46836000 -1.56593600

C 0.99113500 3.94608800 -1.86100900

C -1.39745900 4.09615200 -2.18531500

C 1.17844600 4.97119100 -2.77572400

H 1.85318900 3.49818200 -1.37552600

C -1.20595800 5.13178300 -3.08931800

H -2.40709900 3.80064100 -1.91855000

C 0.08133900 5.56599200 -3.39681000

H 2.18287900 5.31540600 -3.00042500

H -2.06569500 5.61314300 -3.54432500

H 0.22851700 6.37587800 -4.10397700

C 0.29982000 3.46837600 1.56592500

C -0.99109700 3.94614600 1.86099000

C 1.39750000 4.09613800 2.18530400

C -1.17838000 4.97126400 2.77569500

H -1.85316400 3.49826300 1.37550800

C 1.20602800 5.13178200 3.08929700

H 2.40713200 3.80058900 1.91854800

C -0.08125600 5.56603500 3.39678000

H -2.18280200 5.31551200 3.00039000

H 2.06577800 5.61311800 3.54430600

H -0.22841100 6.37593200 4.10394000

C 4.81404500 -1.30070200 0.66268400

H 5.27282900 -1.38396400 1.64669200

C 5.36995300 -1.98655800 -0.37419700

H 4.93800700 -1.90075000 -1.36720600

C -4.81405800 -1.30069100 -0.66267400

H -5.27282600 -1.38397100 -1.64668800

C -5.36998300 -1.98652700 0.37421100

H -4.93805000 -1.90070400 1.36722400

C 6.53731500 -2.82052700 -0.28218300

C 7.04787500 -3.43850200 -1.44843700

C 7.25032600 -3.08351300 0.91282000

C 8.16481200 -4.22928100 -1.40121300

H 6.55924300 -3.29133300 -2.40491900

C 8.36183400 -3.88232400 0.90421900

H 6.93946900 -2.66635800 1.86225900

H 8.57584400 -4.70696000 -2.28246100

H 8.92665900 -4.09536800 1.80389800

C -6.53734600 -2.82049300 0.28219500

C -7.04792300 -3.43845000 1.44845100

C -7.25034200 -3.08349600 -0.91281400

C -8.16486100 -4.22922700 1.40122400

H -6.55930300 -3.29126800 2.40493600

C -8.36185200 -3.88230300 -0.90421600

H -6.93947200 -2.66635600 -1.86225500

H -8.57590600 -4.70689200 2.28247400

H -8.92666700 -4.09535900 -1.80389900

N -8.81901200 -4.45331500 0.23672300

N 8.81897700 -4.45335400 -0.23671700

C 10.04903400 -5.26102200 -0.21860400

H 10.12997600 -5.77172300 0.74095900

H 10.00093400 -6.00673400 -1.01198800

H 10.91637100 -4.61455800 -0.36953700

C -10.04907100 -5.26098000 0.21860500

H -10.12999100 -5.77171000 -0.74094600

H -10.00099200 -6.00666900 1.01201200

H -10.91640900 -4.61451000 0.36949800

(*E*)-TPE-EPy S_0_-geometry：

C -0.41512700 0.73901300 0.21591100

C 0.39146300 -0.35048500 0.19108700

C 0.11331500 2.13161700 0.28278500

C -0.32243000 3.09544100 -0.63361600

C 1.02066600 2.50679600 1.27860300

C 0.16497000 4.39607900 -0.57631500

H -1.04210600 2.81925300 -1.39977900

C 1.49911900 3.81092000 1.34293700

H 1.34895500 1.76989600 2.00586000

C 1.07764500 4.75680400 0.41237800

H -0.17178000 5.13106700 -1.30082700

H 2.19720000 4.09058500 2.12597700

H 1.45086300 5.77489500 0.46291000

C -0.12252400 -1.73943400 0.36129300

C -0.92419200 -2.07814100 1.45610000

C 0.22281600 -2.73622800 -0.55851600

C -1.38908100 -3.37902200 1.61541800

H -1.18033700 -1.31533500 2.18557300

C -0.25141100 -4.03398600 -0.40518000

H 0.86086800 -2.48802200 -1.40261800

C -1.05922000 -4.35833900 0.68244100

H -2.00430800 -3.62976400 2.47408300

H 0.01401300 -4.79502800 -1.13248000

H -1.42154800 -5.37399600 0.80728200

C -1.90061300 0.63363300 0.21132800

C -2.65889100 1.42922100 1.07832600

C -2.57645700 -0.20909100 -0.68539500

C -4.04237200 1.35780000 1.07423800

H -2.15538100 2.10608100 1.76122800

C -3.95708300 -0.27259900 -0.69917900

H -2.00555800 -0.81843100 -1.37742700

C -4.71997200 0.50674300 0.18773500

H -4.60968200 1.97468900 1.76640100

H -4.44111300 -0.93157200 -1.41288300

C 1.86740900 -0.24536000 0.02308100

C 2.71885000 -1.00474600 0.84114100

C 2.43939600 0.55847100 -0.96979100

C 4.09250300 -0.93619700 0.69934500

H 2.28869700 -1.65163200 1.59932600

C 3.81471100 0.61988400 -1.12046100

H 1.79974900 1.14097500 -1.62362000

C 4.66958700 -0.11910900 -0.28756600

H 4.71579000 -1.52750500 1.36250900

H 4.23639500 1.24949300 -1.89967400

C 6.10416400 -0.00618500 -0.49408500

H 6.38408000 0.67102700 -1.29978400

C 7.08174400 -0.63976600 0.19309900

H 6.82103300 -1.32284000 0.99634800

C 8.49867400 -0.49227800 -0.05182900

C 9.41668900 -1.23056600 0.72598300

C 9.06242800 0.35312400 -1.03409300

C 10.76830800 -1.12058100 0.52134100

H 9.06439500 -1.90302300 1.49987800

C 10.42066500 0.42607600 -1.19782400

H 8.44440700 0.96211700 -1.68165000

H 11.49083700 -1.67674500 1.10628800

H 10.88036700 1.06609000 -1.94151800

C -6.17281700 0.48586400 0.22652900

H -6.60533100 1.16357000 0.96114200

C -7.00214600 -0.26656200 -0.53203700

H -6.58912500 -0.95329900 -1.26527000

C -8.44541400 -0.24361100 -0.45486700

C -9.19177100 0.58189500 0.41638500

C -9.19851100 -1.09234200 -1.29472600

C -10.56090200 0.53527200 0.42231800

H -8.70933200 1.26919600 1.09976000

C -10.56921900 -1.09991400 -1.24936100

H -8.70100900 -1.75757900 -1.99124900

H -11.15829900 1.15750500 1.07802400

H -11.16724000 -1.74160300 -1.88505000

N -11.24600100 -0.29652100 -0.39804800

N 11.26766300 -0.30273800 -0.43260800

C -12.71879000 -0.28156000 -0.39217800

H -13.07284800 -0.16229700 0.63203300

H -13.07900400 0.54312800 -1.01077300

H -13.08883000 -1.22743000 -0.78641800

C 12.72276100 -0.16017900 -0.61088000

H 13.07380600 0.71935900 -0.06707300

H 13.21989100 -1.05191000 -0.23044800

H 12.94619200 -0.05632400 -1.67288500

(*E*)-TPE-EPy T_1_-geometry

C -0.45531400 1.77062000 -0.46981100

C 0.39271900 1.46471400 0.72186000

C 0.08509100 2.70910200 -1.45049500

C -0.19701500 2.59117800 -2.82567800

C 0.96621900 3.72826200 -1.03942200

C 0.35093400 3.47783800 -3.74217300

H -0.81939800 1.77438600 -3.17813100

C 1.49791700 4.62177000 -1.95665900

H 1.21705600 3.82650700 0.01294100

C 1.19051400 4.50289800 -3.31144000

H 0.12952300 3.36354700 -4.79862800

H 2.15719000 5.41371600 -1.61596800

H 1.61286100 5.19889900 -4.02904700

C -0.08231100 1.93235600 2.02106000

C -0.88066100 3.09013900 2.10468800

C 0.17901600 1.22034100 3.20858800

C -1.35075800 3.54357600 3.32765500

H -1.11518500 3.64475300 1.20062500

C -0.30742300 1.66949500 4.42835200

H 0.73458600 0.28855100 3.16479000

C -1.06393600 2.83767100 4.49536100

H -1.94592600 4.45002500 3.37230100

H -0.10362600 1.10089100 5.33025200

H -1.43836400 3.19023600 5.45100200

C -1.74939900 1.18558400 -0.56252700

C -2.77950500 1.73207900 -1.37882400

C -2.09124500 0.05265900 0.23404300

C -4.03540300 1.17371500 -1.40481600

H -2.58773800 2.63229600 -1.95082300

C -3.34299100 -0.50720500 0.19171800

H -1.33588600 -0.38401900 0.87908700

C -4.35894300 0.03762400 -0.62995000

H -4.80437300 1.63097300 -2.02214300

H -3.54455100 -1.37904600 0.80578100

C 1.63274700 0.79296200 0.52351400

C 2.70097200 0.86487000 1.46574900

C 1.88640000 0.08206100 -0.68261900

C 3.90999700 0.26076800 1.22671600

H 2.57591000 1.45478300 2.36627200

C 3.09402100 -0.53477900 -0.90683600

H 1.10610600 0.01633400 -1.43353200

C 4.14428100 -0.46693300 0.03622600

H 4.69845500 0.37264000 1.96392800

H 3.24735200 -1.08176700 -1.83351400

C 5.39496800 -1.10960400 -0.27014100

H 5.41878600 -1.60550500 -1.23934100

C 6.51009600 -1.14746900 0.50925900

H 6.50197100 -0.66181900 1.48071900

C 7.74860000 -1.78724900 0.15590100

C 8.83580700 -1.74774800 1.06034300

C 7.98575400 -2.47494200 -1.05889100

C 10.03180300 -2.34271200 0.75819800

H 8.73667500 -1.24259300 2.01441200

C 9.20090900 -3.05254000 -1.31071400

H 7.21946700 -2.56474900 -1.81838900

H 10.87767000 -2.32206000 1.43473200

H 9.40917800 -3.58187500 -2.23284100

C -5.69767900 -0.48415800 -0.69926900

H -6.34993000 0.05358900 -1.38552100

C -6.20926000 -1.54020700 -0.00953600

H -5.57681600 -2.08558600 0.68510700

C -7.56311700 -2.01446300 -0.11026300

C -8.55395300 -1.45596700 -0.95422200

C -7.97830000 -3.11431600 0.67679600

C -9.82252400 -1.96883200 -0.97965200

H -8.34121200 -0.61167200 -1.59755900

C -9.26163300 -3.58860800 0.61270100

H -7.28173800 -3.59893200 1.35144300

H -10.59837100 -1.56048300 -1.61629700

H -9.59982900 -4.42912000 1.20658700

N -10.18018800 -3.02197600 -0.20473100

N 10.21651400 -2.99272800 -0.41518300

C -11.54174100 -3.57268200 -0.29692900

H -12.25138100 -2.75663900 -0.43531600

H -11.60238700 -4.26600400 -1.13867100

H -11.78134000 -4.09505000 0.62886500

C 11.52491300 -3.58068200 -0.74405800

H 12.12302900 -2.85825900 -1.30380000

H 12.03943800 -3.85046400 0.17793600

H 11.37513600 -4.48067300 -1.34070600

**Supplementary References**

1. Chen, X., et al. A Pyridinyl-functionalized Tetraphenylethylene Fluorogen for Specific Sensing of Trivalent Cations. *Chem. Commun.* **49**, 1503-1505 (2013).
